# PLK1 inhibition dampens NLRP3 inflammasome–elicited response in inflammatory disease models

**DOI:** 10.1172/JCI162129

**Published:** 2023-11-01

**Authors:** Marta Baldrighi, Christian Doreth, Yang Li, Xiaohui Zhao, Emily Warner, Hannah Chenoweth, Kamal Kishore, Yagnesh Umrania, David-Paul Minde, Sarah Thome, Xian Yu, Yuning Lu, Alice Knapton, James Harrison, Murray Clarke, Eicke Latz, Guillermo de Cárcer, Marcos Malumbres, Bernhard Ryffel, Clare Bryant, Jinping Liu, Kathryn S. Lilley, Ziad Mallat, Xuan Li

**Affiliations:** 1 The Victor Phillip Dahdaleh Heart and Lung Research Institute, Department of Medicine, University of Cambridge, Cambridge, United Kingdom.; 2 Department of Cardiovascular Surgery, Zhongnan Hospital of Wuhan University, Wuhan, China.; 3 Cancer Research UK Cambridge Centre and; 4 Department of Biochemistry, Cambridge Centre for Proteomics, University of Cambridge, Cambridge, United Kingdom.; 5 Institute of Innate Immunity, University Hospital, University of Bonn, Bonn, Germany.; 6 Cell Division and Cancer Group, Spanish National Cancer Research Centre (CNIO), Madrid, Spain.; 7 Cell Cycle and Cancer Biomarkers Group, “Alberto Sols” Biomedical Research Institute (IIBM-CSIC), Madrid, Spain.; 8 UMR7355 INEM, Experimental and Molecular Immunology and Neurogenetics CNRS and Université d’Orleans, Orleans, France.; 9 Department of Veterinary Medicine, University of Cambridge, Cambridge, United Kingdom.; 10 Université Paris Cité, PARCC, INSERM, Paris, France.

**Keywords:** Cell Biology, Immunology, Cellular immune response, Cytoskeleton

## Abstract

Unabated activation of the NLR family pyrin domain–containing 3 (NLRP3) inflammasome is linked with the pathogenesis of various inflammatory disorders. Polo-like kinase 1 (PLK1) has been widely studied for its role in mitosis. Here, using both pharmacological and genetic approaches, we demonstrate that PLK1 promoted NLRP3 inflammasome activation at cell interphase. Using an unbiased proximity-dependent biotin identification (Bio-ID) screen for the PLK1 interactome in macrophages, we show an enhanced proximal association of NLRP3 with PLK1 upon NLRP3 inflammasome activation. We further confirmed the interaction between PLK1 and NLRP3 and identified the interacting domains. Mechanistically, we show that PLK1 orchestrated the microtubule-organizing center (MTOC) structure and NLRP3 subcellular positioning upon inflammasome activation. Treatment with a selective PLK1 kinase inhibitor suppressed IL-1β production in in vivo inflammatory models, including LPS-induced endotoxemia and monosodium urate–induced peritonitis in mice. Our results uncover a role of PLK1 in regulating NLRP3 inflammasome activation during interphase and identify pharmacological inhibition of PLK1 as a potential therapeutic strategy for inflammatory diseases with excessive NLRP3 inflammasome activation.

## Graphical abstract

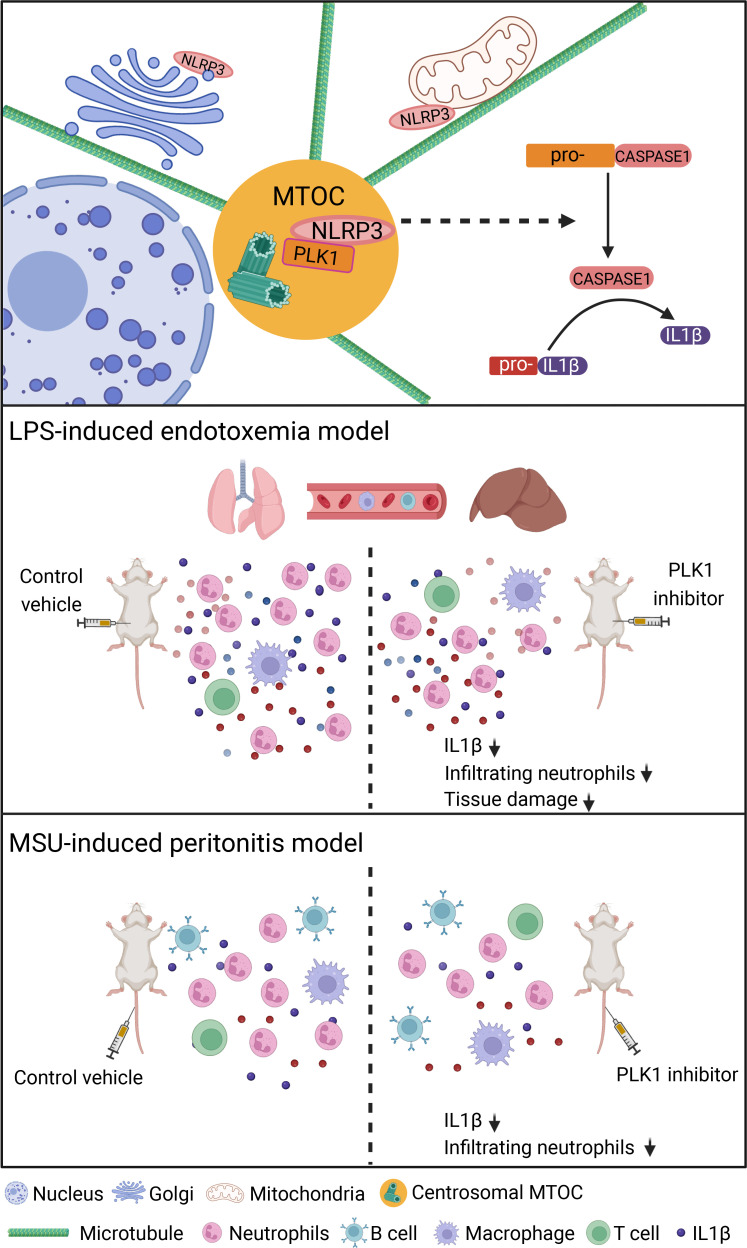

## Introduction

NLR family pyrin domain–containing 3 (NLRP3) inflammasome activation is tightly coordinated by collaborative action of pattern recognition receptors (PRRs), and its excessive activation is associated with various diseases including cryopyrin-associated periodic syndromes, gout, atherosclerosis, diabetes, and Alzheimer’s disease (1–5). Activation of TLRs primes the NLRP3 pathway to enhance basal transcriptional expression levels of NLRP3 and IL-1β (6). Once primed, the cells are then ready to respond to various stimuli that activate the NLRP3 inflammasome to form a multiprotein complex (6), which will result in the production of active IL-1β and IL-18 and pyroptosis (7). The multiprotein complex is formed with NLRP3 connected to caspase 1 through an adaptor protein named apoptosis-associated speck-like protein with a caspase recruitment domain (ASC), which is encoded by *Pycard*. Activation of the NLRP3 inflammasome pathway triggers caspase 1–dependent processing of immature IL-1β (pro–IL-1β) and IL-18 (pro–IL-18) into their bioactive counterparts. Previous studies showed that NLRP3 recruitment to a dispersed *trans*-Golgi network (TGN) is required for NLRP3 aggregation upon activation (8), that the association of cytosolic oxidized mitochondrial DNA with the NLRP3 complex is required for its activation (9), and that NLRP3 positioning at the microtubule-organizing center (MTOC) is required for speck formation (10, 11). Those data demonstrate the importance of correct subcellular localization of NLRP3 for optimal activation of the pathway. Nevertheless, how the tightly controlled NLRP3 inflammasome output is exquisitely coordinated in the different subcellular locations is still largely unknown.

Polo-like kinases (PLKs) are a family of serine/threonine protein kinases that are broadly expressed (12). PLK1 is widely studied for its role in mitosis (13, 14). In recent years, it has become apparent that mitotic proteins can play versatile nonmitotic roles (15–17). The major MTOC in the eukaryotic cell is the centrosome, which consists of a pair of centrioles surrounded by a pericentriolar matrix (PCM) that controls microtubule organization by mediating microtubule nucleation and anchoring (18). PCM undergoes a drastic increase in size during centrosome maturation by accumulating γ-tubulin and other PCM proteins (18, 19), and PLK1 is involved in the recruitment of γ-tubulin and other proteins to PCM, thus regulating centrosome maturation (20–22). However, it is unclear whether PLK1 also plays a similar role in nonmitotic cells. To date, only few studies have indicated a potential role for PLKs in the regulation of inflammation (23–27), and the relevant cellular and molecular mechanisms have remained elusive. For example, Yang et al. showed that PLK4 suppresses NLRP3 inflammasome activation by specifically phosphorylating NEK7 during the LPS-mediated priming step, which in turn inhibits the interaction of NEK7 with NLRP3 and inflammasome activation (27). It is known that PLK1 and PLK4 work together to coordinate mitosis (28, 29). However, it remains unclear whether other PLK proteins are also involved in regulating NLRP3 inflammasome output and, in particular, whether PLKs play a role in the NLRP3 inflammasome activation step at cell interphase.

Here, we revealed the role of interphase PLK1 upon NLRP3 inflammasome activation. We demonstrated that PLK1 was expressed throughout the cell cycle and was detectable during interphase in murine macrophages. Using both pharmacological and genetic approaches, we provided substantial evidence that PLK1 inhibition markedly reduced NLRP3 inflammasome output. Using an unbiased proteomics approach based on proximity-dependent biotin identification (Bio-ID), we revealed the PLK1 interactome in the context of NLRP3 inflammasome activation and show an increased association of NLRP3 with PLK1. We further confirmed that PLK1 interacted with NLRP3 at the endogenous level and identified the interacting domains between these 2 proteins. Mechanistically, we showed that PLK1 reinforced the MTOC structure and regulated NLRP3 subcellular positioning. Most important, low doses of PLK1 kinase inhibitor markedly reduced IL-1β level in both LPS-induced endotoxemia and monosodium urate–induced (MSU-induced) peritonitis models in vivo. This study pinpoints a readily available therapeutic strategy for limiting excessive activation of the NLRP3 pathway in inflammatory diseases.

## Results

### Interphase PLK1 inhibition reduces IL-1β levels upon NLRP3 inflammasome activation.

**Figure 1:**
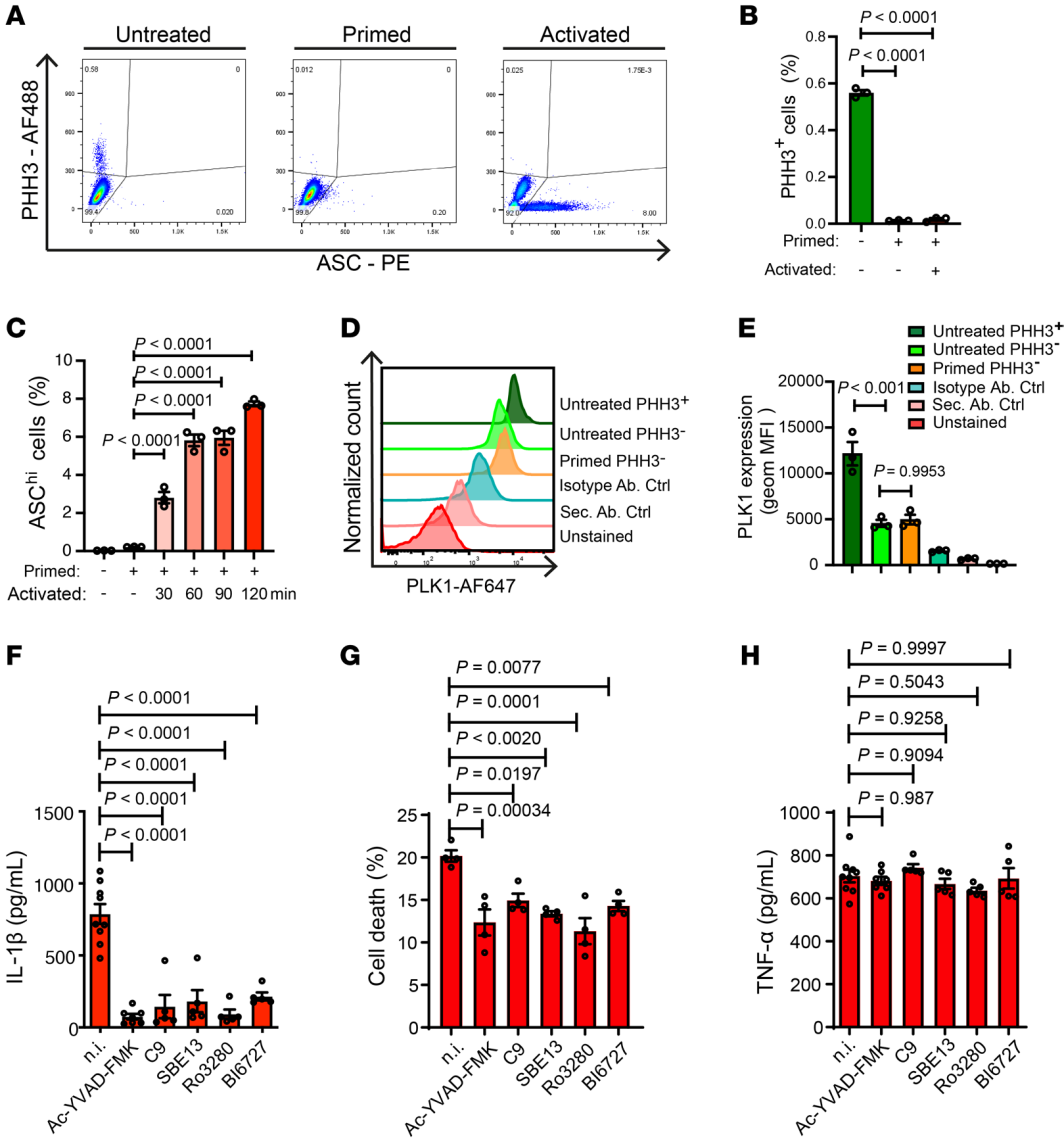
PLK1 inhibition reduces IL-1β output upon NLRP3 inflammasome activation during interphase. (**A**–**C**) After 7 days of differentiation, murine BMDMs were primed (100 ng/mL LPS, 5 hours) and activated (5 μM nigericin, for up to 2 hours). Phosphohistone H3 (PHH3) was used as a mitotic marker (**A** and **B**), and cells with high ASC fluorescence (ASC^hi^) were identified as an activated speck-forming subpopulation (**A** and **C**). *n* = 3/group. PE, phycoerythrin. (**D** and **E**) Geometric (geom) MFI of PLK1 was measured in mitotic cells (dark green) and in nonmitotic cells under the untreated condition (green) and in nonmitotic cells under the primed condition (orange). An isotype antibody control (Isotype Ab. Ctrl) and a secondary antibody control (Sec. Ab. Ctrl) were used. *n* = 3/group. (**F**–**H**) Primed (100 ng/mL LPS, 5 hours) BMDMs were activated (5 mM ATP, 30 minutes), with or without selective PLK1 inhibitors (3 μM cyclapolin 9 [C9]; 10 nM SBE13; 50 nM Ro3280; 0.8 nM BI6727) at the activation stage. The supernatants were collected to measure the IL-1β concentration (**F**), cell death (**G**), and the TNF-α concentration (**H**). The caspase 1 inhibitor Ac-YVAD-FMK or no inhibitor (n.i.) treatment was used as a control. Results are representative of 4 (**A**–**E**) or 3 (**F**–**H**) independent experiments. *n* = 9, 7, 5, 5, 5, and 5 (in order from the left to the right bars in **F** and **H**). *n* = 4/group (**G**). One-way ANOVA with Tukey’s post hoc test was used for statistical analysis. All data are the mean ± SEM.

We used differentiated murine bone marrow–derived macrophages (BMDMs) from WT C57BL/6 mice and found that mitotic cells (identified as phosphohistone H3–positive [PHH3^+^]) represented only a very low percentage (average, 0.56%), which was further ablated after priming with LPS (Figure 1, A and B). Therefore, we confirmed that the primed BMDMs we used for studying inflammasome activation were at the cell interphase. As expected, inflammasome activation significantly enhanced the cell population with a high ASC fluorescence (30) (ASC^hi^) in live cells of WT BMDMs (Figure 1, A and C). To precisely interrogate PLK1 expression in the different cell subpopulations before and after priming NLRP3 activation, we assessed the intracellular expression level of PLK1 by flow cytometry as validated (Supplemental Figure 1A; supplemental material available online with this article; https://doi.org/10.1172/JCI162129DS1). As expected, PLK1 was highly expressed in the mitotic cells before priming, and its level was significantly reduced in nonmitotic cells before and after priming (Figure 1, D and E). PLK1 was expressed throughout the cell cycle and was detectable during interphase in murine macrophages.

The importance of PLK1 in regulating mitosis has led to the successful development of a number of specific PLK1 kinase inhibitors aimed at retarding tumor growth (31). Facilitated by the availability of these specific PLK1 inhibitors (cyclapolin 9 [ref. 32]; SBE13 [ref. 33]; Ro3280 [ref. 34]; BI6727 [ref. 35]), we tested the effect of PLK1 inhibition on IL-1β output upon NLRP3 inflammasome activation stimulated by ATP in WT BMDMs (Figure 1, F–H). PLK1 inhibitors were used at the activation stage (after priming) during the very short time window of ATP stimulation (30 minutes), with caspase 1 inhibitor (Ac-YVAD-FMK) applied as a positive control. Evidently, PLK1 inhibition by all of these compounds substantially reduced IL-1β production (Figure 1F) and cell death (Figure 1G) without affecting TNF-α levels (Figure 1H). We did not observe these inhibitory effects on IL-1β and cell death in NLRP3-deficient cells (Supplemental Figure 1B) or PLK1-deficient cells (Supplemental Figure 1C), indicating that the effect of PLK1 inhibition observed here depended on NLRP3 and PLK1. We detected no effect of PLK1 inhibition on *Il1b* mRNA levels, even when BI6727 was applied during the priming stage (Supplemental Figure 1D). Collectively, these data indicate that PLK1 inhibition during interphase affected NLRP3 inflammasome activation.

### PLK1 promotes NLRP3 inflammasome activation.

**Figure 2:**
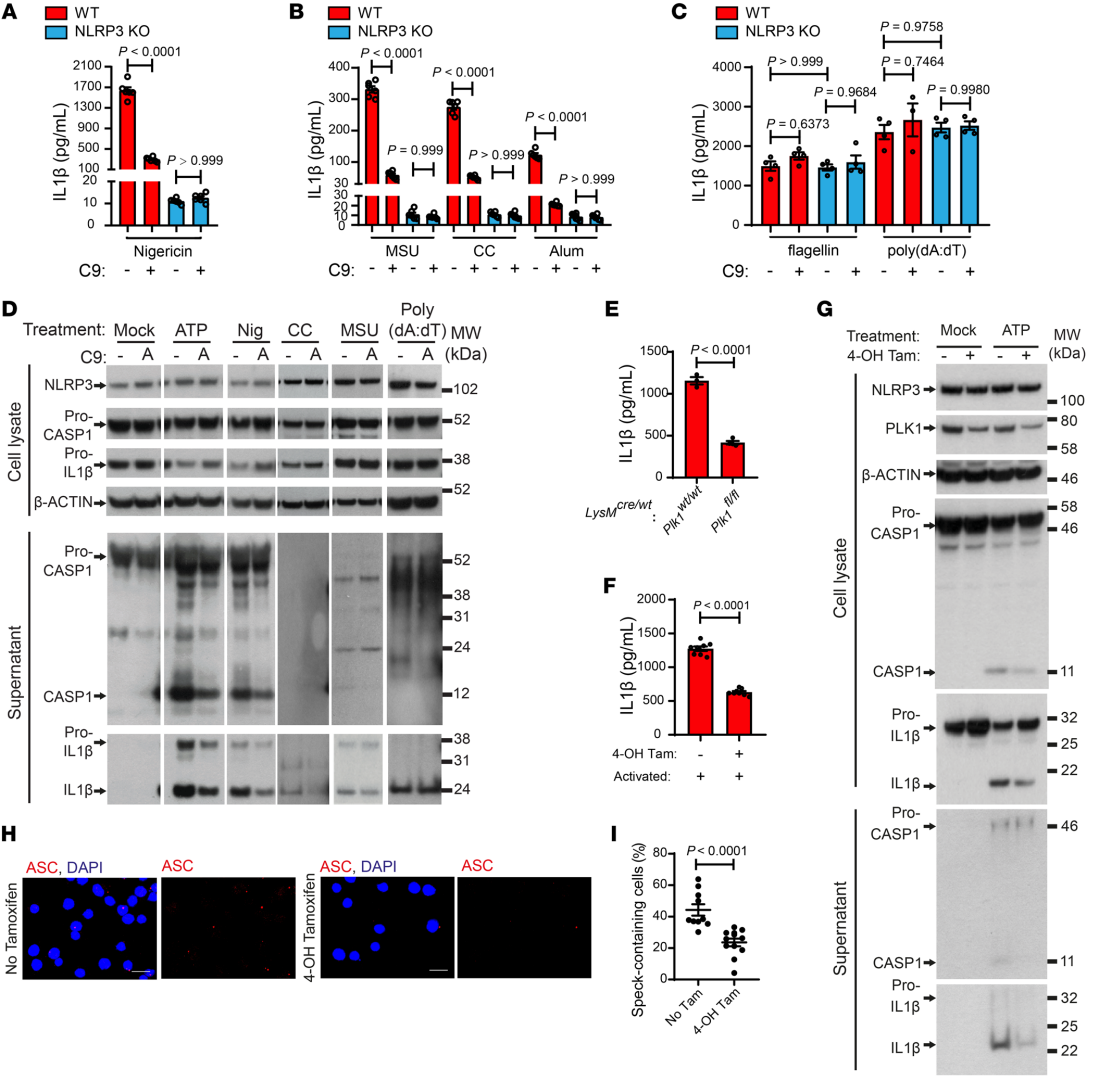
PLK1 inhibition reduces pro–caspase 1 and pro–IL-1β cleavage and ASC speck formation in response to various NLRP3 inflammasome stimuli. (**A**–**C**) Primed (100 ng/mL LPS, 5 hours) WT or NLRP3-KO BMDMs, treated with NLRP3 inflammasome–activating stimuli as indicated (5 μM nigericin for 2 hours; 250 μg/mL MSU for 3 hours; 250 μg/mL cholesterol crystals [CC] for 3 hours; 250 μg/mL Alum crystals for 6 hours; 1 μg/mL flagellin for 3 hours; 1 μg/mL poly(dA:dT) for 3 hours), were subjected to PLK1 inhibition by cyclapolin 9 (3 μM) at the activation stage. Supernatants were collected for IL-1β quantification by ELISA. *n* = 6/group (**A** and **B**); *n* = 3–4/group (**C**). (**D**) Cell lysates and supernatants from WT BMDMs treated as in **A**–**C** as indicated were analyzed by Western blotting. (**E**) Peritoneal macrophages were elicited upon i.p. treatment with 1 mL 3% thioglycolate for 4 days and then isolated. Isolated macrophages were primed (100 ng/mL LPS, 5 hours) and activated (5 mM ATP, 30 minutes). Supernatants were collected for IL-1β quantification by ELISA. Control: *LysM^Cre/WT^* *Plk1^WT/WT^* (*n* = 3); deletion: *LysM^Cre/WT^* *Plk1^fl/fl^* (*n* = 4). (**F**–**I**) *Rosa^CreErt2/WT^* *Plk1^fl/fl^* BMDMs were treated with 4-OH tamoxifen (0.002 mg/mL 4-OH Tam, 24 hours) before and during priming (100 ng/mL LPS, 5 hours) to deplete PLK1, and then cells were activated (5 mM ATP, 30 minutes). (**F**) Supernatants were collected for IL-1β quantification by ELISA (*n* = 8). (**G**) Cell lysates and supernatants were analyzed by Western blotting. Cells stained for ASC were used to quantify the percentage of speck-containing BMDMs across treatments. Scale bars: 20 μm (**H**) and quantification (No Tam, *n* = 10; 4-OH Tam, *n* = 12) (**I**). Results are representative of 3 (**A** and **C**) or 2 (**D**–**G**) independent experiments. Two-way ANOVA with Šidák’s post hoc test was used for statistical analysis (**A**–**C**). An unpaired *t* test was used for statistical analysis (**E**, **F**, and **I**). All data are the mean ± SEM. CASP1, caspase 1; MW, molecular weight.

To confirm the role of PLK1 in regulating the NLRP3 inflammasome pathway, we first used the immortalized BMDMs (iBMDMs) stably expressing ASC-mCerulean (36, 37), in which there was evident speck structure formation after inflammasome activation, as confirmed by flow cytometry (Supplemental Figure 2A). Upon NLRP3 inflammasome activation by nigericin, treatment with a PLK1 kinase inhibitor induced a significant reduction of speck-containing cells, together with reduced IL-1β levels when compared with samples without PLK1 inhibitor treatment (Supplemental Figure 2, B–D). We confirmed and extended these data using BMDMs with a wide set of NLRP3 stimuli, including nigericin and sterile crystals such as MSU crystals, cholesterol crystals (CCs), and aluminium hydroxide (15). PLK1 inhibitor treatment significantly reduced IL-1β production in response to all of those stimuli (Figure 2, A, B, and D). However, the PLK1 inhibitor had no effect on IL-1β levels in response to NLRC4 or AIM2 inflammasome stimuli (e.g., flagellin, dA:dT) (Figure 2, C and D), indicating a specific role of PLK1 in NLRP3 inflammasome activation. We further confirmed that PLK1 inhibition substantially reduced caspase 1 and IL-1β cleavage following NLRP3 inflammasome activation using Western blotting (Figure 2D). These data demonstrate that PLK1 inhibitors are effective in suppressing NLRP3 inflammasome activation.

To delineate the specificity of PLK1 pharmacological inhibitors, we knocked down PLK1 in the myeloid cell lineage by using the LysM Cre recombinase system (38) and confirmed the efficiency of this system in suppressing PLK1 expression in peritoneal macrophages (Supplemental Figure 3, A and B). To account for the possibility that *Plk1^–/–^* peritoneum-resident macrophages derived from the LysM Cre system may undergo impaired differentiation and proliferation during development, we tested the CD11b^+^F4/80^+^ macrophages purified from this procedure (Supplemental Figure 3C) and plated an equal number of cells from all biological replicates. We show that knockdown of PLK1 (indicated as *LysM^Cre/WT^* *Plk1^fl/fl^*) in peritoneal macrophages significantly reduced IL-1β production upon ATP-induced NLRP3 inflammasome activation in comparison with control cells (indicated as *LysM^Cre/WT^* *Plk1^WT/WT^*) (Figure 2E). To further verify our findings, we also applied a tamoxifen-inducible Cre recombination system using Rosa-CreErt2 (39). We confirmed the efficiency of this system in depleting PLK1 expression (Supplemental Figure 3, D–F). Fully-differentiated BMDMs from the *Rosa^CreErt2/WT^* *Plk1^fl/fl^* genetic background were treated with tamoxifen to knock out PLK1 and were then primed with LPS. As shown, *Plk1* depletion did not affect the levels of *Il1b* and *Tnfa* RNA expression (Supplemental Figure 3, H and I). Upon activation with ATP, the *Plk1^fl/fl^* genetic background itself had no effect on IL-1β production (Supplemental Figure 3G). However, we observed a significantly reduced level of IL-1β (Figure 2F) in *Plk1*-depleted BMDMs, with no significant effect on TNF-α production (Supplemental Figure 3J). The effect of *Plk1* depletion on inhibition of NLRP3 inflammasome activation was further confirmed by decreased caspase 1 and IL-1β cleavage on Western blots (Figure 2G) and reduced levels of ASC speck formation (Figure 2, H and I, and Supplemental Figure 3K). Thus, we were able to recapitulate the effect of PLK1 inhibition on NLRP3 inflammasome activation by knocking out PLK1 in 2 independent genetic depletion models. Overall, our results using both pharmacological and genetic inhibition of PLK1 highlight the important role of PLK1 in NLRP3 inflammasome activation.

### Proximity proteomics reveals the PLK1 interactome upon NLRP3 inflammasome activation.

**Figure 3:**
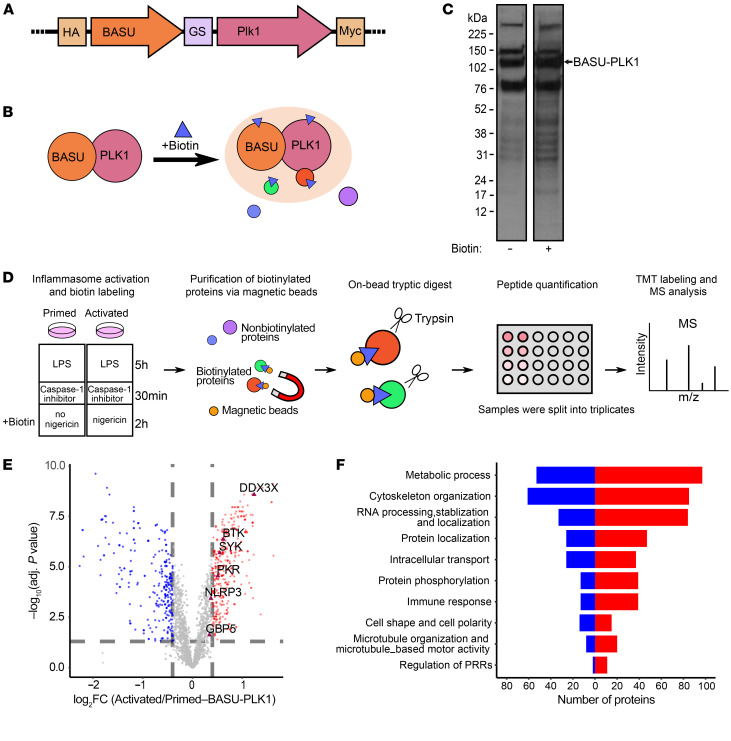
An unbiased Bio-ID screen of PLK1 interactome upon NLRP3 inflammasome activation reveals a proximal association of PLK1 with NLRP3. (**A**) Schematic representation of the bioengineered plasmid expressing the biotin ligase BASU connected to murine PLK1 with a (GGGS)3 linker. (**B**) Schematic representation of the biotinylated proteins associated with PLK1 and BASU in this assay. (**C**) Transduced iBMDMs were treated with biotin (50 μM, 2 hours), and cell lysates were run on a Western blot to show biotinylated proteins stained by streptavidin-HRP. The size of the fusion protein BASU-PLK1 is marked by an arrow. (**D**) iBMDMs transduced with BASU-GS3-PLK1 were treated for inflammasome activation and biotin labeling as indicated. Cells were lysed, and biotinylated proteins were purified using magnetic beads. Trypsinization was followed by peptide quantification, and 5 μg peptides per sample were submitted for TMT labeling and mass spectrometric analysis. (**E**) Volcano plot for the interactome with PLK1 after NLRP3 inflammasome activation compared with the interactome with PLK1 under the primed condition. Red dots represent the enhanced protein interaction in the activated group, blue dots represent the enhanced protein interaction in the primed group, and gray dots are nonsignificant relative to the selected cutoff threshold (the cutoff threshold for the log_2_ fold change [FC] is 0.4, equal to a complete 1.3-fold change; the significance-adjusted *P* value is less than 0.05, by Benjamini-Hochberg correction). (**F**) GO analysis shows the upregulated (red) and downregulated (blue) proteins interacting with PLK1 after NLRP3 inflammasome activation in protein subgroups with corresponding numbers.

To investigate how PLK1 promotes NLRP3 inflammasome activation, we used an unbiased screen based on proximity-dependent Bio-ID (40, 41), which relies on the fusion of a promiscuous biotin ligase to the bait of interest. Here, we used BASU (41), an engineered *Bacillus subtilis*–derived biotin ligase, which we fused to PLK1 (Figure 3A). In our experiment, proteins that came into proximity of the bait protein PLK1 were biotinylated by the biotin ligase BASU, and then the biotinylated proteins were enriched with streptavidin bead pulldown to study the PLK1 interactome (Figure 3, B–D). The BASU enzyme covalently labels lysine residues at its proximity at a rapid speed (41), so this experiment allows the study of protein interaction and even the weak and/or transient interactions in their native states (40). Furthermore, covalent biotinylation enables harsh lysis conditions without losing this modification. Therefore, Bio-ID also allows the identification of protein interactions at the organelles, such as the cytoskeleton, nuclear envelope (40), and centrosome (42), which are refractory to conventional methods for studying protein interactions. To validate whether ectopically expressed BASU-tagged PLK1 could retain native PLK1 function, we reconstituted chemical/genetic PLK1-KO telomerase-expressing human retinal pigment epithelial (tRPE) PLK1 analog-sensitive (PLK1^AS^) cells (43, 44) with BASU-tagged PLK1 and then induced cell-cycle arrest introduced through PLK1 deletion by 3MB-PP1 treatment as described previously (43, 44). PLK1^AS^ refers to a mutant tRPE cell line, which express mutant PLK1^AS^ with an altered catalytic pocket that can bind bulky purine analogs. The addition of these purine analogs (3MP-PP1) will make a genetic/chemical inhibition that completely interrupts PLK1 function in these cells in a highly efficient manner with practically no off-target effects, as the analogs are too large to fit into the catalytic pockets of other endogenous kinases (43). Our data showed that BASU-tagged PLK1 performed as well as the HA-tagged PLK1 or endogenous PLK1 in rescuing cell arrest (Supplemental Figure 4).

To identify the PLK1 interactome in the context of NLRP3 inflammasome activation, we transduced iBMDMs and established a macrophage cell line that stably expressed BASU-tagged PLK1. LPS priming (100 ng/mL for 5 hours) was applied to these cells before biotin addition. Given the presence of biotin trace in the culture medium (45), cells expressing BASU-tagged PLK1 had basal level of biotinylation even without adding more biotin, however, we observed increased intensity of biotinylated proteins after biotin addition (Figure 3C). Prior to nigericin activation of the NLRP3 inflammasome, cells were preincubated with the caspase 1 inhibitor Ac-YVAD-cmk to reduce cell death. The samples from cells with nigericin treatment were used to identify the altered PLK1 interactome after NLRP3 inflammasome activation (activated), and the samples from cells with no nigericin treatment were used as controls (primed). After purification of the biotinylated proteins, equal amounts of the total peptides (5 µg) were subjected to tandem mass tag (TMT) labeling and liquid chromatography tandem mass spectrometry (LC-MS/MS) (Figure 3D).

In total, 1,252 candidates passed quality control (Supplemental Figure 5A and Supplemental Table 1.1–1.3). After batch normalization, principal component analysis (PCA) revealed that the experimental groups were correctly clustered, and the activated samples were clearly distinguished from the primed samples (Supplemental Figure 5B and Supplemental Table 1.4). Most important, the mass intensity of the identified interactors with PLK1 (normalized by PLK1 value in each batch) was evidently elevated after NLRP3 inflammasome activation (Supplemental Figure 5C and Supplemental Table 1.5), suggesting expanded protein complex formation with PLK1 caused by NLRP3 inflammasome activation. After removing the candidates that responded to BASU tagging alone, the remaining candidates were split on the volcano plot to show the fold change of their proximal association with PLK1 after NLRP3 inflammasome activation (Figure 3E and Supplemental Table 1.3). A significant group of candidates involved in the immune response, including the PRR signaling pathway, showed an increased association with PLK1 after inflammasome activation (Figure 3F and Supplemental Table 2). Among these candidates, several previously known NLRP3 interactors, including DDX3X (46), BTK (47), SYK (48, 49), PKR (50), GBP5 (51), and NLRP3 itself, were identified as proximal interactors with PLK1 upon NLRP3 activation (Figure 3E and Supplemental Table 1.3).

PLK1 is a central player involved in regulating cytoskeletal architecture during mitosis (52). In the context of inflammasome activation, we found that candidates involved in cytoskeleton organization constituted a major group of proteins interacting with PLK1 (Figure 3F). To delineate these interactions in further detail, we analyzed subcellular localization of the interacting proteins (Supplemental Table 1.6). We found that, of all the cytoskeletal players, proteins involved in microtubule and centrosome organization showed overall significantly increased interaction with PLK1 (Supplemental Figure 5, D and E, and Supplemental Table 1.7). These results strongly indicate a role of PLK1 in organizing MTOC upon NLRP3 inflammasome activation.

### PLK1 interacts with NLRP3.

**Figure 4:**
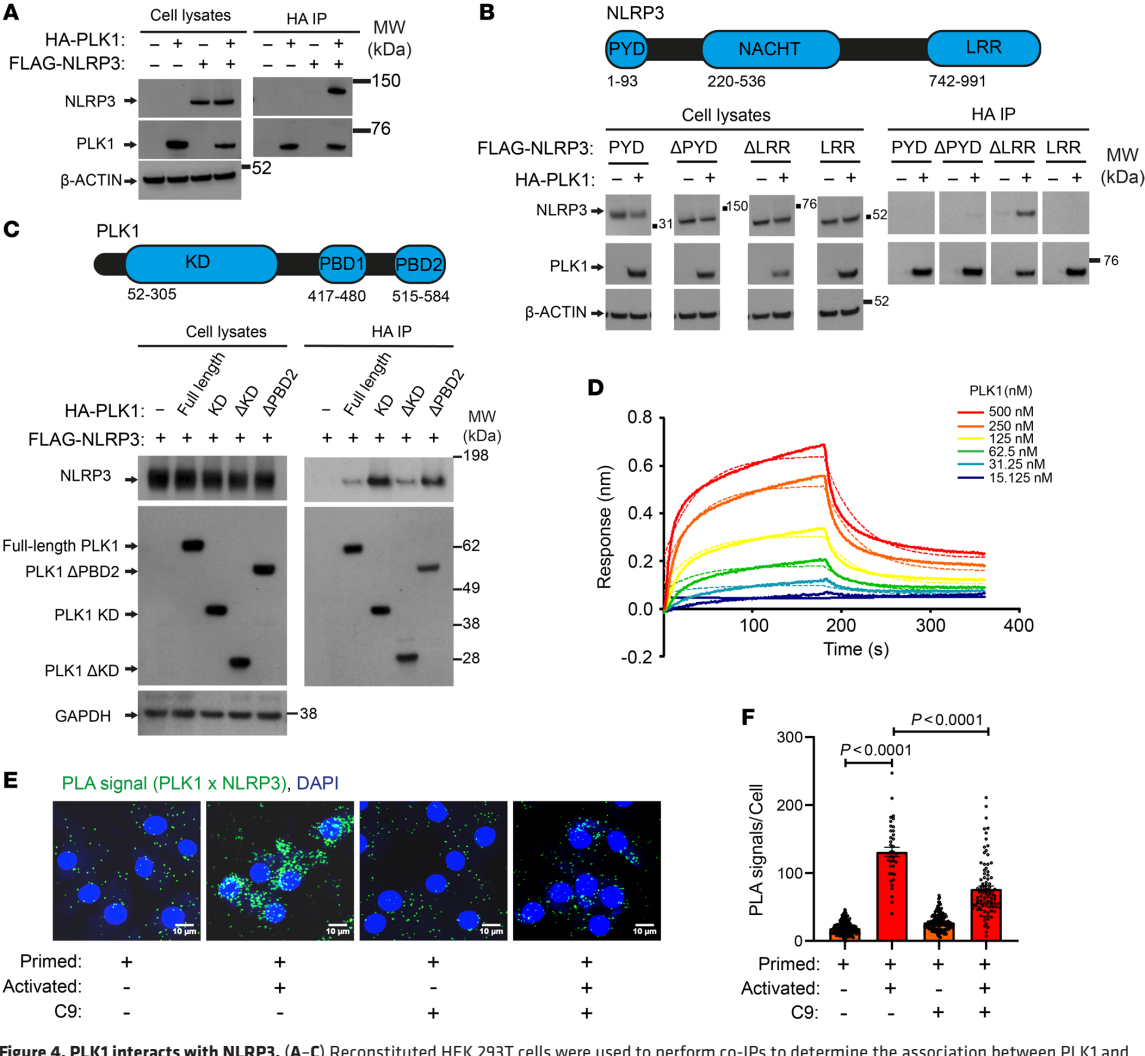
PLK1 interacts with NLRP3. (**A**–**C**) Reconstituted HEK 293T cells were used to perform co-IPs to determine the association between PLK1 and NLRP3 using full-length proteins (**A**), NLRP3 domains (PYD, pyrin domain; ΔPYD, pyrin domain deletion; LRR, leucine-rich repeat; ΔLRR, LRR deletion) with full-length PLK1 (**B**), or PLK1 domains (ΔKD, KD deletion; ΔPBD2, PBD2 deletion) with full-length NLRP3 (**C**). Domain structures of NLRP3 and PLK1 are shown in **B** and **C**. Whole-cell lysates were analyzed as an indication of transfection. (**D**) Bio-Layer interferometric analysis with immobilized, purified NLRP3 protein as the ligand and purified PLK1 protein as the analyte of different concentrations. (**E** and **F**) BMDMs were primed (100 ng/mL LPS, 5 hours) and then activated (5 mM ATP, 30 minutes). PLK1 inhibition with 3 μM cyclapolin 9 was used at the activation stage. Interaction between PLK1 and NLRP3 was detected by PLA. Scale bars: 10 μm (**E**), and quantification of PLA signals per cells (*n* = 185, 38, 150, and 88, in order from the left bar to the right bar) (**F**). Two-way ANOVA with Šidák’s post hoc test was used for statistical analysis. All data are the mean ± SEM.

PLK1 consists of N-terminal Ser/Thr kinase domain (KD) and conserved C-terminal polo-box domains (PBDs) (53, 54). Whereas KD is important in phosphorylating its substrates, phospho-dependent ligand recognition by PBD is necessary for PLK1 to target the specific substrates and subcellular locations (54). To further validate and delineate the PLK1-NLRP3 proximal association identified in the PLK1 interactome, we induced co-overexpression of HA-tagged PLK1 together with Flag-tagged NLRP3 in HEK293T cells, in which the endogenous inflammasome proteins are lacking (55). We confirmed that PLK1 coimmunoprecipitated with NLRP3 (Figure 4A). To understand the molecular interaction between PLK1 and NLRP3, we mapped this interaction using various truncations of these 2 proteins and found that full-length PLK1 interacted with pyrin-NACHT domains of NLRP3 (Figure 4B). We also found that full-length NLRP3 interacted with both the KD and PBD of PLK1 (Figure 4C), suggesting that both the kinase activity and ligand-binding function of PLK1 were required for its association with NLRP3. Furthermore, we demonstrated that purified recombinant PLK1 protein (full-length) was able to bind to immobilized recombinant NLRP3 protein (full-length) using a Bio-Layer interferometry assay (Figure 4D), suggesting a direct binding between these 2 proteins is plausible.

An in situ proximity ligation assay (PLA) is suitable for quantitative studies of endogenous protein expression, protein modifications, and close protein interactions (56, 57). Using PLA to detect protein-protein interaction in situ, we confirmed that PLK1 was associated with NLRP3 at the endogenous level in BMDMs (Figure 4, E and F). Furthermore, this PLK1-NLRP3 interaction was enhanced after NLRP3 inflammasome activation, and this increased interaction was susceptible to PLK1 kinase inhibition (Figure 4, E and F). Hence, our results confirm that PLK1 interacts with NLRP3 and that NLRP3 inflammasome activation can increase PLK1 kinase activity, which further promotes PLK1-NLRP3 interaction. In the activated samples with PLK1 kinase inhibition, PLK1-NLRP3 interaction did not drop to the same level as in primed-only samples, consistent with the data that PBD was also required for its optimal interaction with NLRP3.

### PLK1 reinforces MTOC organization and regulates NLRP3 subcellular positioning.

**Figure 5:**
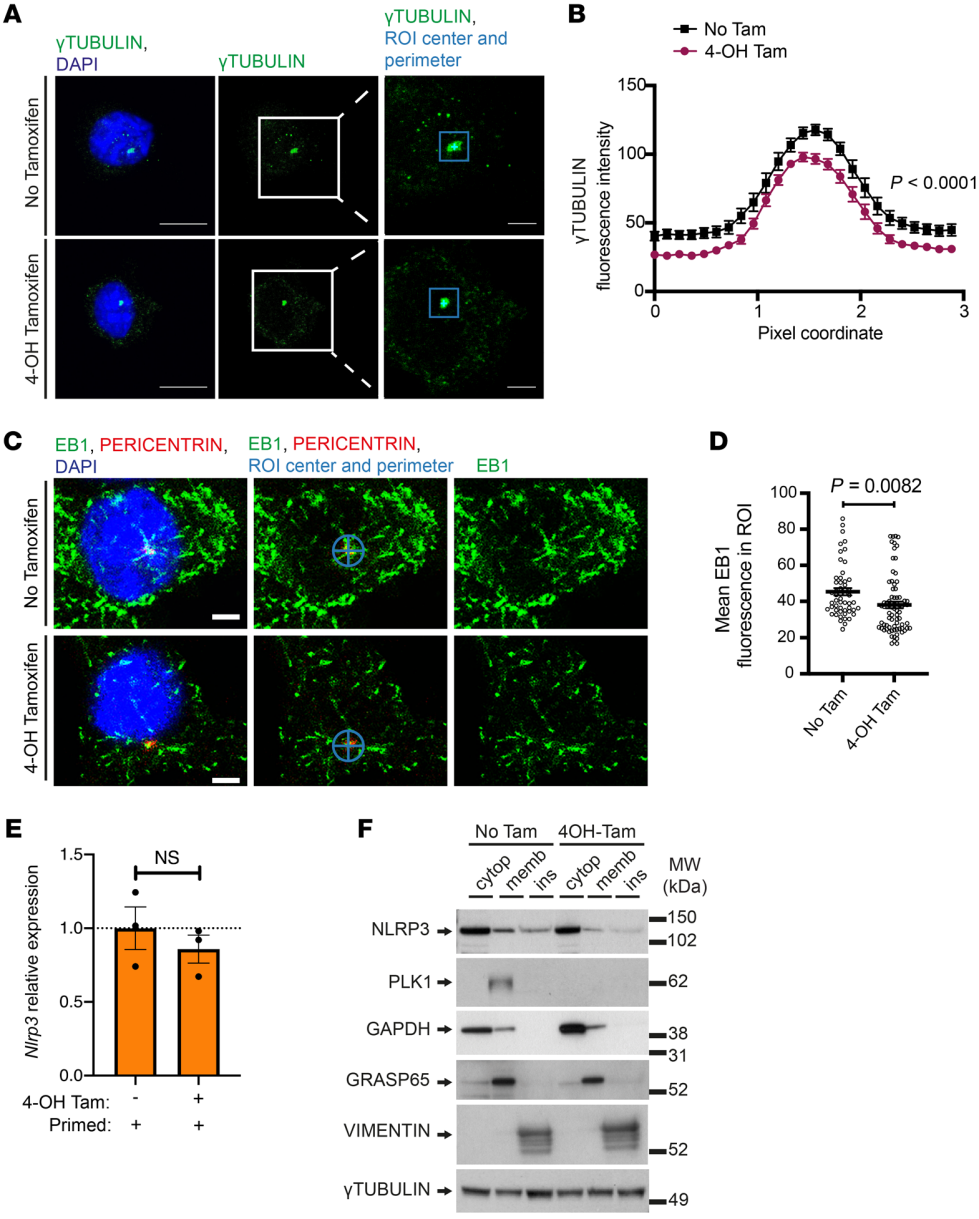
PLK1 regulates microtubule nucleation and affects NLRP3 inflammasome positioning. (**A** and **B**) *Rosa^CreErt2/WT^* *Plk1^fl/fl^* BMDMs were treated with 4-OH Tamoxifen (0.002 mg/mL 4-OH Tam, 24 hours) before and during priming (100 ng/mL LPS, 5 hours) and were then activated (5 mM ATP, 30 minutes). γ-Tubulin fluorescence was quantified within a 3 × 3 μm ROI centered around the main γ-tubulin focus. Scale bars: 10 μm (lower magnification) and 3 μm (enlarged insets) (**A**). Quantification by fluorescence intensity (*n* = 20) (**B**). (**C** and **D**) *Rosa^CreErt2/WT^* *Plk1^fl/fl^* BMDMs were treated as in **A**. EB1 fluorescence was quantified in a circular ROI of 3 μM diameter. Scale bars: 3 μm (**C**). Quantification by mean EB1 fluorescence (No Tam, *n* = 54; 4-OH Tam, *n* = 77) (**D**). (**E**) *Rosa^CreErt2/WT^* *Plk1^fl/fl^* BMDMs were treated with 4-OH tamoxifen (0.002 mg/mL 4-OH Tam, 24 hours) before and during priming (100 ng/mL LPS, 5 hours). *Nlrp3* relative expression was quantified by qPCR. *n* = 3/group. (**F**) *Rosa^CreErt2/WT^* *Plk1^fl/fl^* BMDMs were treated as in **A** and processed for fractionation Western blotting. cytop, cytoplasmic; memb, membrane; ins, insoluble fractions. GAPDH, GRASP65, and vimentin, respectively, were used as markers for each fraction. (**F**) Results are representative of 2 independent experiments. (**A**–**F**) Treatments without 4-OH tamoxifen were used as controls. Two-way ANOVA with Šidák’s post hoc test (**B**) and an unpaired *t* test (**D** and **E**) were used for statistical analysis. All data are the mean ± SEM.

Since PLK1 influences the recruitment of γ-tubulin complexes to the mitotic centrosomes (21), we asked if PLK1 would regulate MTOC structural composition upon inflammasome activation during interphase. In the primed and activated cells with *Plk1* depletion (tamoxifen-induced PLK1 depletion in *Rosa^CreErt2/WT^* *Plk1^fl/fl^* BMDMs), we analyzed γ-tubulin content at the centrosomal MTOC and measured microtubule growth by tracking microtubule plus end tip end binding 1 (EB1) growing at the microtubule minus end. In the absence of PLK1, we observed a significant reduction of centrosomal γ-tubulin accumulation (Figure 5, A and B), along with a reduction of microtubule growth from the centrosome as indicated by EB1 (Figure 5, C and D).

Microtubule-associated transport is the key intracellular machinery involved in regulating protein subcellular localization (58). To further test whether PLK1 could influence NLRP3 subcellular localization, we used a biochemical fractionation assay. As shown, there were comparable levels of *Nlrp3* expression in these cells (Figure 5E). However, in comparison with cells with no PLK1 depletion upon NLRP3 inflammasome activation, we detected a reduced presence of NLRP3 in both membrane and insoluble cytoskeletal fractions of the PLK1-depleted cells, despite no apparent changes in the cytosolic fractions (Figure 5F). These results suggest altered NLRP3 trafficking between different cellular organelles. Overall, our data highlight the role of PLK1 in organizing MTOC and the microtubule network in the NLRP3 inflammasome pathway and the role of PLK1 in influencing the distribution of its binding partner NLRP3 to specific subcellular locations that are likely associated with optimal NLRP3 inflammasome activation.

### PLK1 kinase inhibition suppresses IL-1β levels in inflammatory models in vivo.

**Figure 6:**
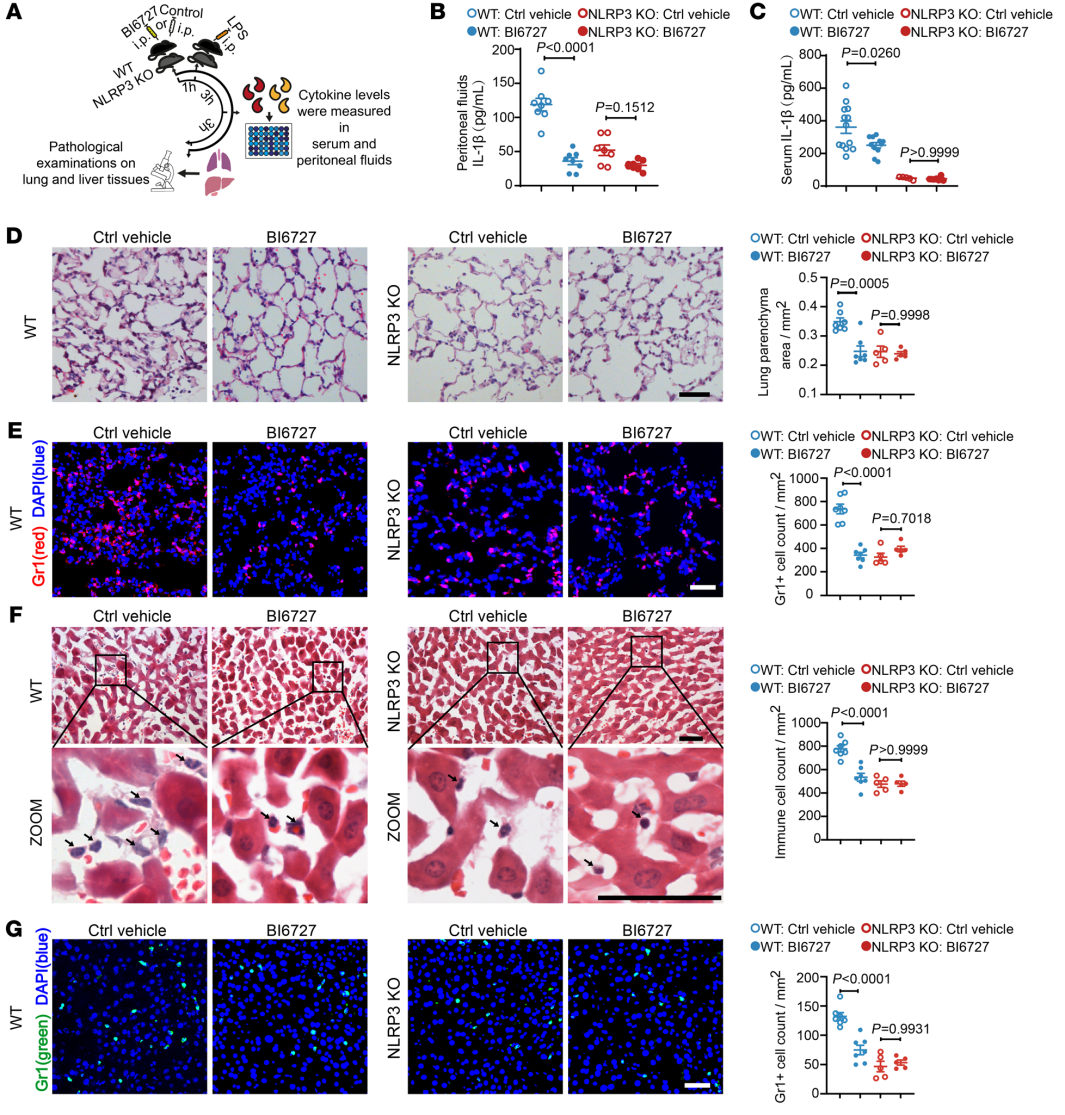
PLK1 inhibition suppresses the inflammatory response in a LPS-induced endotoxemia model. C57BL/6 WT and NLRP3-KO mice were treated with BI6727 (5 mg/kg, i.p.) or control (Ctrl) vehicle, followed by LPS administration (20 mg/kg, i.p.). (**A**) Experimental scheme. Samples for cytokine measurement and tissue assessment were collected at the indicated time points. (**B** and **C**) IL-1β levels were measured in peritoneal fluids (**B**) (WT: Ctrl vehicle, *n* = 8; WT: BI6727, *n* = 8; NLRP3-KO: Ctrl vehicle, *n* = 7; NLRP3-KO: BI6727, *n* = 8), and in serum (**C**). WT: Ctrl vehicle, *n* = 13; WT: BI6727, *n* = 11; NLRP3-KO: Ctrl vehicle, *n* = 5; NLRP3-KO: BI6727, *n* = 7. (**D** and **E**) Representative histopathological images from lung tissues and quantification of lung parenchymal area (**D**), and representative immunofluorescence images of Gr1^+^ cell staining in lung tissue and quantification (**E**). WT: Ctrl vehicle, *n* = 7; WT: BI6727, *n* = 7; NLRP3-KO: Ctrl vehicle, *n* = 5; NLRP3-KO: BI6727 group, *n* = 5. (**F** and **G**) Representative histopathological images of the liver and quantitative results of immune cell infiltration (**F**), and representative immunofluorescence images of Gr1^+^ cells in liver and quantitative results (**G**). WT: Ctrl vehicle, *n* = 7; WT: BI6727, *n* = 7; NLRP3-KO: Ctrl vehicle, *n* = 5; KO: BI6727, *n* = 5. Scale bars: 50 μm. Two-way ANOVA with Šidák’s post hoc test was used for statistical analysis. All data are the mean ± SEM.

PLK1 is essential for cell proliferation and survival during development, and generic *Plk1*-KO mice display an embryonic lethality phenotype (59). Therefore, we applied a widely used pharmacological PLK1 kinase inhibitor, BI6727, at doses of 5 mg/kg i.p. in a LPS-induced endotoxemia model (Figure 6A), or 1 mg/kg i.v. in a MSU-induced peritonitis model. The doses were lower than the doses at which BI6727 is normally used in cancer studies (35). In these two models we have used, IL-1β production was largely or totally dependent on NLRP3 (1, 17, 60).

Upon LPS challenge in the peritoneal cavity, we detected a substantial reduction of IL-1β levels in the mice treated with BI6727 when compared with mice treated with vehicle control (Figure 6, B and C), in both peritoneal fluids and serum. As shown, IL-1β levels were largely dependent on NLRP3 activation in the LPS-induced endotoxemia model, as IL-1β was substantially reduced in NLRP3-KO control mice, regardless of pharmacological treatment (Figure 6, B and C). Under BI6727 treatment, there was only a limited effect of BI6727 on reducing IL-1β levels in the peritoneal fluids of NLRP3-KO mice (Figure 6, B and C), indicating that the effect of BI6727 on IL-1β was largely mediated by NLRP3. However, the effect of BI6727 on reducing the LPS-induced inflammatory response was not solely through its inhibition of IL-1β. For example, we also detected that BI6727 treatment in this inflammatory model led to the reduction of TNF-α in both the peritoneal fluids and serum independently of NLRP3 (Supplemental Figure 6, A and B), consistent with the previously described role of PLK1 in TLR-mediated TNF-α production in monocytes (25).

LPS administration leads to systematic inflammatory responses including inflammation in the lung (61) and liver (62), and the degree of the inflammatory response could largely depend on NLRP3 (11, 63). We showed that BI6727 significantly alleviated LPS-induced inflammatory responses in the lung (Figure 6, D and E) and the liver (Figure 6, F and G), as evidenced by reduced levels of alveolar wall thickness (Figure 6D) and infiltration of immune cells (including Gr1^+^ cells, which are often used to represent neutrophils in the tissues) (64) (Figure 6, E–G). BI6727 did not further limit the inflammatory response in NLRP3-KO mice (Figure 6, D–G), further confirming that the effect of BI6727 on dampening inflammation depended on NLRP3.

**Figure 7:**
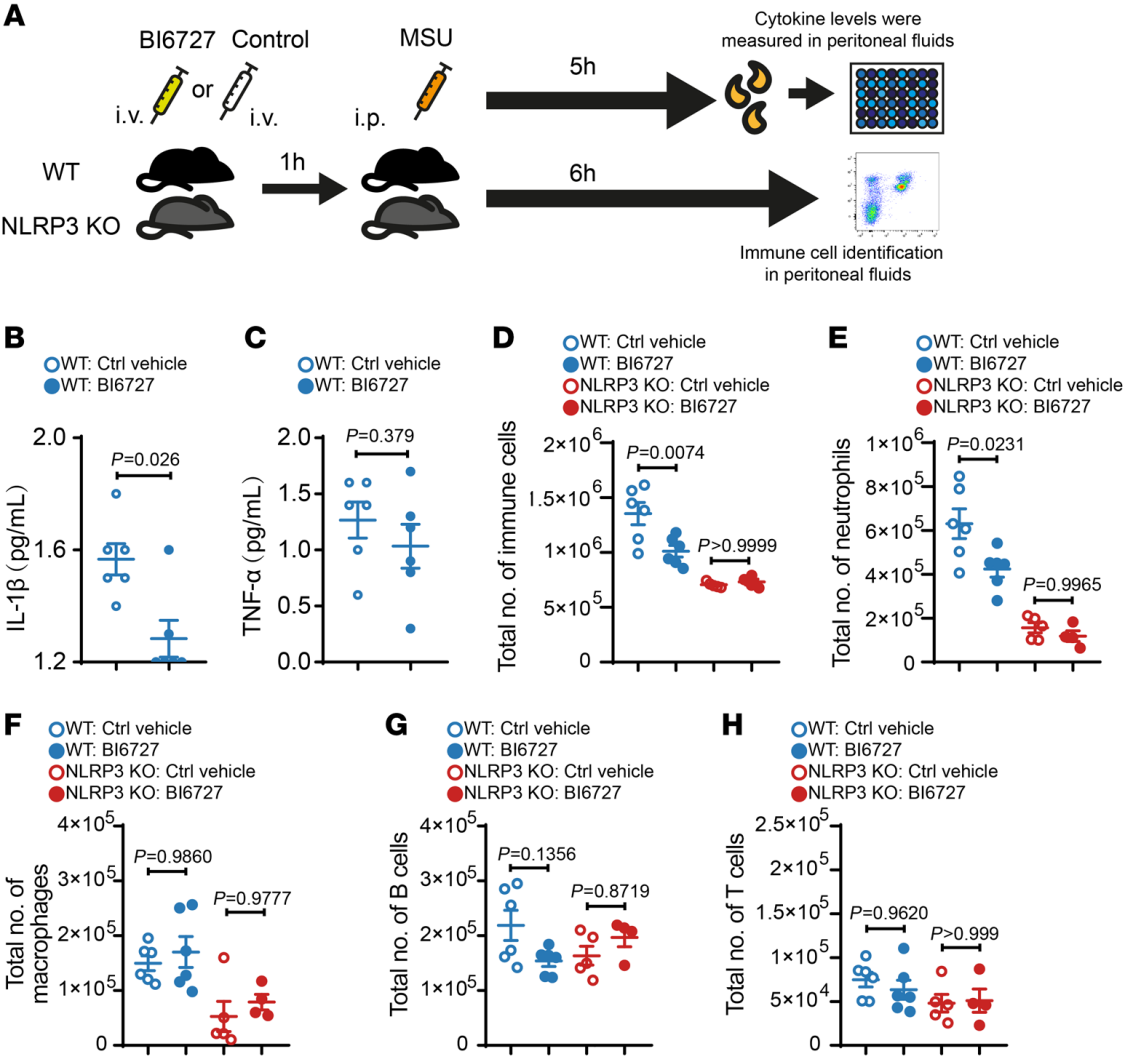
PLK1 kinase inhibition suppresses the inflammatory response in a MSU-induced peritonitis model. C57BL/6 WT mice and NLRP3-KO mice were treated with BI6727 (1 mg/kg, i.v.) or control vehicle, followed by MSU (0.5 mg/mouse, i.p.). (**A**) Experimental scheme. Samples for cytokine measurement and flow cytometry were collected at the indicated time points. (**B** and **C**) IL-1β (**B**) and TNF-α (**C**) levels in peritoneal fluids. *n* = 6/group. (**D**–**H**) Cells collected from the peritoneal cavity were analyzed by flow cytometry. Quantification of total number of immune cells (**D**), neutrophil cells (**E**), macrophages (**F**), B cells (**G**), and T cells (**H**). WT: Ctrl vehicle, *n* = 6; WT: BI6727, *n* = 6; NLRP3-KO: Ctrl vehicle, *n* = 5; NLRP3 KO: BI6727, *n* = 4. Statistical analysis was done by nonparametric test (**B**), unpaired *t* test (**C**), and 2-way ANOVA with Šidák’s post hoc test (**D**–**H**). All data are the mean ± SEM.

To further test the effect of BI6727 in vivo, we also used an MSU-induced peritonitis model (Figure 7A), in which IL-1β levels were completely dependent on NLRP3 activation and the increased IL-1β levels upon MSU treatment led to neutrophil infiltration into the peritoneal cavity. In this model, we showed that IL-1β levels in peritoneal lavage were substantially reduced under BI6727 treatment without significantly altering TNF-α levels (Figure 7, B and C). Furthermore, we showed that BI6727 reduced the number of infiltrated neutrophils (not macrophages, B cells, or T cells) in the peritoneal cavity and that this reduction was dependent on NLRP3 (Figure 7, D–H, and Supplemental Figure 7).

Collectively, our data confirm that PLK1 plays a major role in mediating IL-1β levels and that PLK1 kinase inhibition is effective in suppressing IL-1β during the inflammatory response in vivo.

## Discussion

In mitotic cells, centrosome maturation is prepared to enforce the MTOC structure by recruitment of several PCM components (65, 66), and PLK1 plays an important role in this process (20, 21, 67–69). Here, we show that upon NLRP3 inflammasome activation, interphase cells used a similar mechanism to regulate centrosomal MTOC organization and arrangement and that this process was also controlled by PLK1. PLK1 allowed for optimization of NLRP3 inflammasome activation and subsequent IL-1β production. PLK1 deletion reduced γ-tubulin recruitment at the centrosomal MTOC, leading to stunted microtubule growth. Our data, together with what others have demonstrated (11, 17, 27), strongly indicate that the interphase centrosome serves as a major microtubule-organizing center where the innate immune response is orchestrated. Importantly, we found that the PLK1 kinase inhibitor BI6727, which is currently being tested in oncology clinical trials, was remarkably effective in suppressing IL-1β production when used even at a low dose in 2 independent inflammatory models in vivo. These results suggest a promising therapeutic strategy for repurposing this drug to treat diseases with excessive activation of the NLRP3 inflammasome and enhanced IL-1β production.

Furthermore, in the LPS-induced endotoxemia model, which is not solely dependent on NLRP3 to elicit an inflammatory response, we observed that BI6727 treatment also reduced TNF-α levels (Supplemental Figure 6). Our data, combined with previously described effects of PLK1 on TLR-mediated TNF-α (24, 25) and IFN (23, 24) levels, strongly suggest a broad role of PLK1 in regulating the inflammatory response. PLK1 inhibition could be widely beneficial in suppressing inflammation.

Taken together, our data not only unravel the mechanistic roles of PLK1 in regulating NLRP3 inflammasome activation, but also propose a clinically available strategy for treating NLRP3-dependent inflammatory disorders. Beyond the well-known role of PLK1 in controlling mitosis, we show that PLK1 plays a moonlighting role in interphase to control inflammation. This work expands our current understanding of PLK1 and NLRP3 functions, as well as our knowledge of how the subcellular compartments are possibly modulated by microtubule organization to deliver the inflammatory output.

## Methods

### Mice.

WT C57BL/6 and NLRP3-KO mice were previously described (60). All mice were fully backcrossed onto a C57BL/6 background. Clare Bryant (Veterinary Medicine, University of Cambridge, Cambridge, United Kingdom) and Bernard Ryffel (CNRS, INEM UMR7355, Orleans, France) provided the NLRP3-KO mice. Guillermo de Carcer and Marcos Malumbres provided the *Plk1^fl/fl^* mice (Spanish National Cancer Research Centre, Madrid, Spain). *LysM^Cre^* mice were obtained from The Jackson laboratory. David Adams (Wellcome Sanger Institute, Hinxton, Cambridgeshire, United Kingdom) provided the *Rosa^CreErt2/CreErt2^* mice.

### LPS-induced endotoxemia model.

Age- (8–14 weeks), sex-, and genetic (C57BL/6J) background–matched male WT mice and NLRP3-KO mice were used. Mice fed a chow diet were injected i.p. with 5 mg/kg BI6727 (Selleckem, S2235) resuspended in vehicle control. Vehicle control consisted of 4% DMSO (MilliporeSigma, D5879) in corn oil (MilliporeSigma, C8267). One hour after BI6727 administration, the mice were injected i.p. with 20 mg/kg LPS (MilliporeSigma, O111:B4, L4391) resuspended in PBS. Three hours after LPS injection, the animals were sacrificed, and peritoneal exudates and blood were collected and measured to measure cytokine levels. Six hours after the LPS injection, another batch of mice were sacrificed, and then lung and liver tissue were harvested for analysis.

### MSU-induced peritonitis model.

Age- (8–12 weeks), sex-, and genetic (C57BL/6J) background–matched male WT mice and NLRP3-KO mice were used. Mice fed a chow diet were injected i.v. with 1 mg/kg BI6727 (Selleckem, S2235) resuspended in vehicle control as previously described (35). Vehicle control consisted of 0.01% DMSO in 0.9% NaCl with 0.1N HCl. One hour after BI6727 administration, the mice were injected i.p. with MSU crystals (0.5 mg/mouse, resuspended in 200 μL PBS). Five hours after MSU challenge, the animals were sacrificed, and peritoneal exudates were collected to measure cytokine levels. Six hours after MSU challenge, another batch of mice were sacrificed, and then cells in the peritoneal cavity were collected for flow cytometric analysis.

### Histological analysis of tissue.

Lung and liver tissues were fixed in 4% paraformaldehyde for 24 hours and transferred to 30% sucrose solution in PBS overnight. Tissues were then embedded in OCT compound for cryosectioning. Murine left lung tissue (or tissue from the left lateral hepatic lobe) was evenly divided into 3 equal parts to represent 3 different levels and then cryosectioned into 5 μm slices. H&E staining was performed following previously described procedures (70). The buffers and reagents used were as follows: Harris’ Hematoxylin (MilliporeSigma, HHS128), Scott’s Solution (20 g magnesium sulphate and 2 g sodium bicarbonate were dissolved in 1 liter of distilled water), eosin (MilliporeSigma, HT110280), destaining solution (250 mL methanol, 250 mL filtered H_2_O, and 5 mL concentrated HCl), and DPX (MilliporeSigma, 06522).

The severity of lung injury was assessed by the area of parenchyma. The lung parenchymal area assessed combined the area of the alveolar septum, the area of alveolar exudation, the area of inflammatory cell infiltration, and the area of bleeding. The area was determined on the basis of the measurement using ImageJ software (NIH). For each mouse, the lung parenchymal area measurement was averaged for 4–6 fields per section, 1 section per level, and 3 different levels in total. For each mouse, the infiltrated immune cells in the liver sinusoid were averaged for 4–6 fields per section, 1 section per level, and 3 different levels in total.

### Immunofluorescence.

The cryosectioned tissues were used for immunofluorescence as previously described (71). The images were taken with a Leica microscope (DM6000 B). All analysis was performed using 4–6 fields per section at 1 level (comparable levels among different mice) for each mouse.

Immunofluorescence of cells was acquired on a Leica SP5 confocal microscope. For γ-tubulin quantification, images were acquired as *Z*-stacks with equal steps between each focal plane. Images were analyzed in FIJI (ImageJ). The point-of-interest tool was centered on the brightest γ-tubulin spot for each centrosome in a maximum-intensity *Z* projection image. The point of interest was added to the region of interest (ROI) manager to determine its coordinates, and a square selection was created, adjusting the sides to 3 μm and locating the center on the coordinates of the previously selected point of interest. Fluorescence intensities within the ROI were determined.

EB1 comets were quantified manually in FIJI as previously described (72). Single-plane images focused on the brightest pericentrin signal were marked with a point of interest corresponding to the pericentrin focus. A circular selection of 3 μm in diameter was centered around the point of interest. EB1 fluorescence intensity was measured within the circular selection. For each randomly selected field of view, all focused centrosomes were analyzed.

### Flow cytometric analysis.

The peritoneal lavage samples were collected and centrifuged, and the total number of cells was counted using a NucleoCounter cell counter (Chemometec). Collected cell samples were then stained with Zombie Aqua viability dye (BioLegend, 423102) for 10 minutes at room temperature in the dark. Then, samples were stained with the desired antibodies as previously described (73, 74). Data were analyzed with FlowJo software (version 10.8.1, BD) and the gating strategy provided in Supplemental Figure 7. Total immune cells were defined as cells with the surface marker CD45, excluding debris and gating on singlets to identify live cells. From CD45^+^ cells, the cells within the CD45^+^B220^–^CD4^–^CD8^–^Ly-6C^–^Ly-6G^–^CD11b^+^F4/80^+^ gate represented macrophages. B cells were defined as cells with the B220 surface marker, and T cells were defined as cells with the surface markers CD4 and CD8. The cells within the CD45^+^B220^–^CD4^–^CD8^–^Ly-6C^+^Ly-6G^+^ gate represented neutrophils.

### Plasmids and molecular biology.

Full-length and truncated human NLRP3 (94-979; 1-220; 1-389; 1-574; 575-979) were subcloned into pCMV-3Tag-1 as described (ref. 10). pCMV3-HA-human PLK1 was acquired from Sino Biological (HG10676-NY). Truncations of PLK1 (1-361, 1-498, 362-603) were then cloned into a pCMV5-HA vector. The BASU sequence (Addgene, 107250) was subcloned into pLenti-C-Myc-DDK-P2A-Puro (OriGene, PS100092V5). Full-length murine Plk1 was then subcloned into the resulting plasmid, and a (GGGS)3 linker was inserted between the Plk1 and BASU sequence to create the pLenti-BASU-Plk1-Myc-DDK-P2A-Puro plasmid used for lentiviral transduction. The BASU-Plk1 sequence was further cloned into a pCMV5-HA vector to create pCMV5-BASU-Plk1.

### Antibodies.

Antibodies against NLRP3 (AdipoGen Life Sciences, AG-20B-0014-C100; MilliporeSigma, HPA012878), IL-1β (R&D Systems, AF-401-NA), caspase 1 (Santa Cruz Biotechnology, SC-154), HA tag (Santa Cruz Biotechnology, y-11), Flag tag (MilliporeSigma, F1804), PLK1 (Abcam, ab17056), β-actin (Cell Signaling Technology, 3700S), and ASC (Enzo Life Sciences, AD1-905-173-100) were used for Western blotting. Anti–rabbit IgG-HRP (Santa Cruz Biotechnology, sc-2357), anti–mouse IgG-HRP (Santa Cruz Biotechnology, sc-516102), and anti–goat IgG-HRP (Agilent Technologies, P044901-2) were used for Western blot protein detection with ECL substrate (GE Lifesciences, RPN2106). Antibodies against PLK1 (Proteintech, 10305-1-AP) and NLRP3 (Abcam, ab4207) were used for in situ PLAs. CD45-eVolve 605 (eBioscience, 83-0451-42), B220-APC/cyanine 7 (BioLegend, 103223), CD4–Pacific Blue (BioLegend, 100531), CD8–Pacific Blue (BioLegend, 100725), CD11b–Alexa Fluor 488 (BioLegend, 101217), F4/80–PerCP/cyanine 5.5 (BioLegend, 123128), Ly-6G-PE (BD Biosciences, 551461), Ly-6C–Alexa Fluor 647 (BioLegend, 128010), phosphorylated histone H3 (Ser10) Alexa Fluor 488 (BioLegend, 650804), PLK1 (Proteintech, 10305-1-AP), Alexa Fluor 647 goat anti–rabbit IgG (Invitrogen, Thermo Fisher Scientific, A21246), ASC (BioLegend, 653904), rabbit IgG isotype control (Thermo Fisher Scientific, 02-6102), and AF488 donkey anti–rabbit IgG (Thermo Fisher Scientific, A-21206) were used for flow cytometry. Antibodies against PLK1 (Abcam, ab17056), ASC (Santa Cruz Biotechnology, sc-22514-R), γ-tubulin (MilliporeSigma, T5326), EB1 (BD, 610534), pericentrin (Abcam, ab4448), Gr1 (Invitrogen, Thermo Fisher Scientific, 14-5931-85), Alexa Fluor 488 donkey anti–rat IgG (Invitrogen, Thermo Fisher Scientific, A21208), and Alexa Fluor 555 donkey anti–rat IgG (Invitrogen, Thermo Fisher Scientific, A78945) were used for immunofluorescence. Nuclei were stained with DAPI (MilliporeSigma, D9542).

### Cell culturing.

WT C57BL/6 and *Rosa^CreErt2/WT^* *Plk1^fl/fl^* bone marrow cells were obtained from mice. Bone marrow cells were differentiated into macrophages as previously described (75). BMDM differentiation was confirmed by flow cytometry with the markers CD11b and F4/80. PLK1 depletion in the *Rosa^CreErt2/WT^* *Plk1^fl/fl^* system was carried out by incubation of 0.002 mg/mL 4OH-tamoxifen (H7904-5MG, MilliporeSigma) for 24 hours. ASC-mCerulean iBMDMs (provided by Eicke Latz, University of Bonn, Bonn, Germany) were cultured in RPMI 1640 GlutMax (Thermo Fisher Scientific, 61870044) supplemented with 10% FBS (Gibco, Thermo Fisher Scientific, 10270106) and antibiotics (MilliporeSigma, P4333). HEK293T cells (ATCC) were cultured in DMEM (MilliporeSigma, D6546) supplemented with 10% FBS, 2 mM l-glutamine (MilliporeSigma, G7513), and 1 mM sodium pyruvate (MilliporeSigma, S8636). tRPEs and tRPE PLK1^AS^ cells were provided by Prasad Jallepalli (Sloan Kettering Institute, New York, New York, USA) through Ashok Venkitaraman (MRC Cancer Unit, Cambridge, University of Cambridge, Cambridge, United Kingdom). tRPEs were cultured in DMEM/F12 media with HEPES and l-glutamine (Thermo Fisher Scientific, 11320033) supplemented with 10% FBS and antibiotics. Incubation of tRPE PLK1^AS^ cells overnight with 20 μM of the purine analog 3MP-PP1 (Cayman Chemicals, 17860) was done to induce cell-cycle arrest, and cells in cell-cycle arrest were counted over 3 fields of view. Cells were cultured at 37°C in 5% CO_2_ in a humidified incubator.

### Isolation of Plk1-KO peritoneal macrophages.

To circumvent differentiation-related positive selection of PLK1-expressing macrophages, thioglycolate (MilliporeSigma, T9032; 1 mL sterile 3% w/v PBS-based thioglycolate, i.p.) was used to elicit peritoneal macrophages as previously described (76). Peritoneal fluids were collected after 4 days of thioglycolate treatment by peritoneal lavage from *LysM^Cre/WT^* *Plk1^fl/fl^* and *LysM^Cre/WT^* *Plk1^WT/WT^* male mice on the C57BL/6 background at 12–14 weeks of age. Peritoneal macrophages were purified using the AutoMACS Pro Separator system (Miltenyi Biotec, 130-092-545), and purity was confirmed by flow cytometry with the markers CD11b and F4/80. Cells were cultured in RPMI 1640 GlutaMAX supplemented with 10% FBS and antibiotics for 2 hours prior to priming with LPS and activation with ATP.

### Treatments.

NLRP3 inflammasome priming was performed by incubation with 100 ng/mL or 500 ng/mL LPS (O111:B4, MilliporeSigma, L4391) for 5 hours. Activation was induced by the following treatments, as indicated: 5 mM ATP (MilliporeSigma, A7699) for 30 minutes; 3 μM nigericin (Enzo, BML-CA421-0005) for 2 hours; 5 μM nigericin for up to 2 hours; 10 μM nigericin for 1 hour; 250 mg/mL MSU for 3 hours; 250 mg/mL cholesterol crystals for 3 hours; 250 mg/mL Alum crystals (Thermo Fisher Scientific, 771610) for 6 hours; 1 mg/mL flagellin (Enzo, ALX-522-058-C010) for 3 hours; 1 mg/mL poly (dA:dT) (InvivoGen, tlrl-patn) for 3 hours. MSU crystals and cholesterol crystals were made as described previously (77, 78). BMDMs were treated with the following PLK1 inhibitors: 3 μM cyclapolin 9 (Tocris, 3316), 10nM SBE13 (Selleckchem, S7720), 50nM Ro3280 (Stratech, S7248-SEL), and 0.4 or 0.8 nM BI6727 (Selleckchem, S2235). A CytoTox 96 nonradioactive cytotoxicity assay (Promega, G1780) was used to measure lactate dehydrogenase (LDH) as an indication of cell viability following the manufacturer’s instruction.

### Transfection.

For co-IP, cells were transfected using the CaCl_2_ method as previously described (79). Flagellin and dsDNA poly (dA:dT) were transfected with DOTAP reagent (Roche, 11202375001) to activate inflammasomes. The NeonTM transfection system (Thermo Fisher Scientific, MPK5000) was used for the transfection of tRPE cells with pCMV3-HA-hPLK1 or pCMV5-BASU-GS3-Plk1, according to the manufacturer’s instructions. Cells were then incubated in a cell culture medium without antibiotics overnight before any experiment was conducted.

### Lentivirus transduction.

pLenti-BASU or pLenti-BASU-Plk1 plasmids were used to package and produce lentiviruses by transient transfection of HEK293/T17 cells using the TransIT-LT1 (MirusBio, MIR 2300). iBMDMs were transduced with packaged lentivirus as previously described. Twenty-four to 48 hours after transduction, cells were placed in media without virus, and puromycin selection was performed to establish stable iBMDMs expressing BASU or BASU-PLK1.

### Cell treatment for Bio-ID.

Stable iBMDMs expressing BASU or BASU-PLK1 were primed with 100 ng/mL LPS (MilliporeSigma) for 5–6 hours and/or with 10 μM activated nigericin for 2 hours. Thirty minutes prior to nigericin activation, cells were treated with 100 μM Ac-YVAD-cmk (MilliporeSigma, SML0429) and 5 mM glycine. During nigericin treatment, cells were incubated with 50 μM biotin. After that, both floating and adherent cells were collected in PBS with 1 mM EDTA. The resulting cell pellets were lysed in the lysis buffer (50 mM Tris, pH 7.4, 500 mM NaCl, 0.4% SDS, 5 mM EDTA, 1% Triton X-100) freshly supplemented with 1 mM DTT and 1 tablet of cOmplete Mini EDTA-free Protease Inhibitor Cocktail (Roche, 11836170001) per 10 mL buffer. The lysates were then passed through a 25 gauge syringe 10 times for complete lysis. Then, the samples were sonicated on a Bioruptor Pico (Diagenode; sonication cycle: 30 seconds on, 30 seconds off; 5 cycles, 4°C). Samples were centrifuged at 16,000*g* for 10 minutes at 4°C, and the supernatant was collected and frozen at –80°C for storage. Samples were then desalted using a PD-10 Desalting Column (GE Healthcare, 52130800) according to the manufacturer’s instructions.

### Biotinylated protein purification.

The protocol for biotin purification was adapted from Schopp et al. (80) and Seidi et al. (81). Pierce Streptavidin Magnetic Beads (Thermo Fisher Scientific, 8816) were equilibrated (50 mM Tris, pH 7.4, 150 mM NaCl, 0.05%Triton X-100). Up to 4 mg proteins (measured with the DC protein assay) were incubated with 200 μL equilibrated beads, and the mixture was incubated overnight with rotation at 4°C. The beads were then washed in 4 different washing buffers in sequence for 8 minutes each on a rotating wheel at room temperature (wash buffer 1: 2% SDS; wash buffer 2: 50 mM HEPES, pH 7.4, 1 mM EDTA, 500 mM NaCl, 1% Triton X-100, 0.1% nadeoxycholate; wash buffer 3: 50 mM Tris, pH 7.4, 1 mM EDTA, 250 mM LiCl, 0.5% P-40, 0.5% Na-deoxycholate; wash buffer 4: 50 mM Tris, pH 7.4, 50 mM NaCl, 0.1%NP-40). Bead-bound proteins were then treated with reducing solution (10 mM DTT in 100 mM triethylammonium bicarbonate [TEAB]) for 30 minutes at 56°C, followed by alkylation in 55 mM iodoacetamide in 100 mM TEAB for 45 minutes at room temperature in the dark. Beads were then washed in TEAB buffer for 15 minutes on a rotating wheel. Trypsin digestion was performed overnight at 37°C with 20 ng/μL MS grade trypsin (Thermo Fisher Scientific, 90057) in 50 mM TEAB, pH 8. Peptides (5 μg) for each sample in triplicate were submitted to the Cambridge Centre of Proteomics for further processing.

### TMT labeling and mass spectrometric data analysis.

For TMT labeling, the TMTsixplex Isobaric Label Reagent Set (Thermo Fisher Scientific) was used. Briefly, the submitted peptides were dried with a SpeedVac vacuum concentrator (Thermo Fisher Scientific) and resuspended in 50 μL TEAB buffer, pH 8. Before use, the TMT label reagents were brought to room temperature and resuspended in acetonitrile, and 0.4 mg TMT label reagents were added to each peptide sample. After incubation at room temperature for 1 hour, the reaction was stopped with the addition of 8 μL 5% hydroxylamine for 15 minutes. The labeled samples were combined and cleaned using Pierce C18 Spin Tips (Thermo Fisher Scientific). For LC-MS/MS, the Dionex Ultimate 3000 RSLC nanoUHPLC (Thermo Fisher Scientific) system and a Lumos Orbitrap mass spectrometer (Thermo Fisher Scientific) were used. The peptides were loaded onto a pre-column (Thermo Fisher Scientific, PepMap 100 C18, 5 μm particle size, 100 A pore size, 0.3 mm diameter × 5 mm length) with 0.1% formic acid for 3 minutes at a flow rate of 10 μL/minute. Then, the peptides were eluted onto the analytical column (EASY-Spray Column; Thermo Fisher Scientific, PepMap C18, 2 μm particle size, 100 A pore size, 75 μm diameter × 50 cm length) for separation of peptides via reverse-phase chromatography at a flow rate of 300 nL/minute. A 2 % to 40% linear gradient of 80% acetonitrile in 20% water plus 0.1%formic acid in water plus 0.1% formic acid over 93 minutes was used. The eluted peptides were introduced into the mass spectrometer with an EASY-Spray source (Thermo Fisher Scientific). *m/z* values were measured with an Orbitrap Mass Analyzer (Thermo Fisher Scientific) with a resolution of 120,000 and were scanned between *m/z* 380 and 1,500 Da. Precursor ions were isolated and fragmented by collision-induced dissociation (CID) (normalized collision energy [NCE]: 35%), which were analyzed in the linear ion trap. Synchronous precursor selection was used to select the top 10 most abundant fragment ions from each MS/MS for further fragmentation in the high energy collision cell using high-energy collisional dissociation (HCD) (NCE: 65%). The Orbitrap analyzer measured all *m/z* values, and the relative abundance of each reporter ion and all fragments in each MS step with a resolution at 60,000.

Raw data processing was performed using Proteome Discoverer, version 2.4 (Thermo Fisher Scientific). The data were searched against the Uniprot Mouse database and a database of common contaminants by the Mascot search algorithm (Matrix Science).

Spectra identification was performed with the following parameters: MS accuracy, 10 ppm; MS/MS accuracy, 0.8 Da; up to 2 trypsin missed cleavage sites allowed; carbamidomethylation of cysteine and TMT tagging of lysine and peptide N-terminus as fixed modifications; and oxidation of methionine and deamidated asparagine and glutamine as variable modifications. The Percolator node was used for FDR estimation, and only peptide identifications of high confidence (FDR  <1%) were accepted.

### Bioinformatics analysis of Bio-ID proteomics.

A total of 6,856 proteins were identified from 3 TMT batches and 12 samples. However, only 3,063 of these proteins were consistently identified in all 12 samples. Missing protein values across different samples in 1 TMT batch were relatively low, and no protein with missing values was exclusively present in the primed or activated samples. Missing values were removed from subsequent analysis. Differential expression (DE) analysis was performed to identify interacting proteins that were upregulated and downregulated in activated versus primed cells. There were 60 proteins with low FDR confidence that we removed to keep high-quality proteins in the DE analysis. A total of 3,063 proteins remained after removing the missing value and low FDR. Then, a UniProtKB/Swiss-Prot (reviewed, manually annotated, March 2021) search was applied to filter out the unreviewed proteins in the list. Eventually, 1,252 proteins passed the quality control assessment and were applied for the following analysis (DE, Gene Ontology [GO], and subcellular localization).

First, normalization and batch correction were applied for all samples using the R (version 4.1.2) qPLEXanalyzer (version 1.12.0) package’s “normalizeScaling” function. The central median normalization was used for log-scaled intensities. The “ComBat” function (sva package, version 3.42.0) was used for batch correction. Paired DE analysis identified 247 upregulated proteins and 172 downregulated proteins with a cutoff absolute log_2_ fold change of greater than 0.4 (equal to an absolute 1.3-fold change) and a Benjamini-Hochberg–corrected adjusted *P* value (adj.P.Val) of less than 0.05. The total differentially interacting proteins with statistical significance (419) were used for GO unbiased analysis with clusterProfiler (version 4.2.2, R package). Subcellular localization analysis used the data from The Human Protein Atlas (https://www.proteinatlas.org/humanproteome/subcellular), and mouse proteins were converted to human proteins and then matched to the main location. Given the mouse/human orthology, 999 of 1,252 genes were assigned with human subcellular localization. To check the interacting protein with PLK1 at the subcellular location between primed and activated samples, density plot and *P* value analyses were performed for the selected categories.

### In situ PLA.

Cells were cultured on a collagen-coated (MilliporeSigma, 125-50) 8-well chamber slide (Thermo Fisher Scientific, 177402). The PLA assay was performed according to the manufacturer’s instructions (Duolink). Live cells were fixed with 4% PFA and then subjected to the PLA assay. Images were acquired with a Carl Zeiss LSM 700 microscope. PLA signals per cytoplasm of a cell were acquired using Duolink analysis software.

### Co-IP assay.

Cell lysates from HEK293T cells were collected and lysed in NP40 lysis buffer (500 mM Tris HCl, pH 8, 500 mM MgCl_2_, 2.5 M NaCl, 0.25 M EGTA, pH 8, 5% NP40) supplemented with 1 mM Na_3_VO_4_ and 50 mM NaF and 1 tablet of cOmplete, Mini, EDTA-free Protease Inhibitor Cocktail per 10 mL buffer (Roche). For co-IP using the HA-tag, proteins were incubated with rabbit anti-HA antibody (Santa Cruz Biotechnology, sc-805) for 2 hours, rotating at 4°C followed by overnight incubation with M-280 sheep anti–rabbit IgG Dynabeads (Thermo Fisher Scientific, 11203D). Beads were isolated using a magnetic block and washed 5 times with washing buffer (50 mM Tris HCl, pH 7.4, 250 mM NaCl), rotating at 4°C for 30 minutes before they were collected in 1× NuPAGE LDS Sample Buffer (Thermo Fisher Scientific, NP0007) supplemented with 1:100 DTT and stored at –20°C.

### Biolayer interferometry.

To analyze protein-protein interactions, biolayer interferometry experiments were performed using the Octet RED96 (FortéBio) instrument at 25°C and 1,000 rpm. Recombinant full-length NLRP3 tagged with GST (Caltag Medsystems, H00114548), diluted in assay buffer (100 mM HEPES, 100 mM NaCl, pH 7.4, 0.01%BSA, 0.01% Tween-20) to 7.2 nM, was immobilized on anti-GST–coated fiber optic biosensors (FortéBio). A blank anti-GST–coated biosensor was used as a control reference. Immobilized biosensors were then suspended in buffer containing recombinant full-length PLK1 (tagged with His; MRC-PPU) at 500–15.125 nM for 180 seconds to measure the association phase and then transferred to buffer for 180 seconds to measure the dissociation phase. Biosensors were regenerated between each reading using 10 mM glycine (pH 2.0) for 5 seconds and neutralized in assay buffer for 5 seconds; this was repeated 3 times for each cycle. Subtraction of reference sensor data, and Savitsky-Golay filtering and kinetics parameter derivation were conducted in FortéBio Data Analysis software (version 8.0). Data were graphed using GraphPad Prism (GraphPad Software). Nonspecific binding of PLK1 to empty anti-GST biosensors and recombinant GST alone was negligible (data not shown).

### Western blotting.

Supernatants of cells treated with inflammasome-activating stimuli were collected in serum-free media. TCA protein precipitation was performed on supernatants to detect cleaved IL-1β and caspase 1. TCA protein precipitation was performed on those supernatants to detect cleaved IL-1β and caspase 1. Cell lysates were recovered in Triton X-100 lysis buffer (20 mM Tris HCl, pH 8, 5 mM MgCl_2_, 10 mM EGTA pH 8, 1% Triton X-100). DSS cross-linking and speck detection in insoluble protein fractions was performed as previously described (82). Protein samples were separated using precast NuPAGE Novex 4% to 12% Bis-Tris gels (Invitrogen, Thermo Fisher Scientific, NP0336BOX), and proteins were transferred onto iBlot PVDF membranes (Thermo Fisher Scientific, 88585). Detection of antibodies was performed using an ECL substrate (GE Lifesciences, 28980926). Fractionation Western blotting was performed using the Subcellular Protein Fractionation Kit (Thermo Fisher Scientific, 87790) according to the manufacturer’s instructions. See complete unedited blots in the supplemental material.

### Quantitative PCR.

RNA was extracted from BMDMs using the RNeasy Plus Mini Kit (QIAGEN, 74106) according to the manufacturer’s instructions. cDNA was generated using the QuantiTect Reverse Transcription Kit (QIAGEN, 205313) according to the manufacturer’s instructions. Quantitative PCR (qPCR) reactions were run on a LightCycler machine (Roche) using Mesa Green qPCR Mastermix (Eurogentec, 10-SY2X-03+NR WOU) according to the manufacturer’s instructions.

### ELISA and Meso Scale Discovery.

Mouse IL-1β ELISA kits were obtained from BD Biosciences (catalog 559603) and Thermo Fisher Scientific (catalog 88-7013-88), and mouse TNF-α was obtained from R&D Systems (catalog DY410). Mouse IL-6 was obtained from Thermo Fisher Scientific (catalog 88-7064-88), and mouse IL-12 p70 was obtained from Thermo Fisher Scientific (catalog 88-7121-88). ELISAs were performed according to the manufacturer’s instructions. Meso Scale Discovery (MSD) assays were performed by the core biochemical assay laboratory of Cambridge University Hospitals. The detection limit of mouse IL-1β by MSD was 1.2 pg/mL.

### Statistics.

Statistical analysis was performed with GraphPad Prism. Data are expressed as the mean ± SEM in all plots. Comparisons of the 2 different groups were analyzed by 2-tailed, unpaired *t* test or by paired *t* test as indicated. For more than 2 groups, 1-way ANOVA followed by Tukey’s test (for 1 variable) and 2-way ANOVA with Šidák’s multiple-comparison test (for 2 independent variables and multiple groups) were used. When data did not follow a normal distribution, nonparametric tests were used to analyze the data. A *P* value of less than 0.05 was considered statistically significant.

### Study approval.

The animal studies were performed following UK Home Office regulations, under approved Home Office project licenses PPL PA4BDF775 and PP9485757 (United Kingdom).

### Data availability.

All bioinformatics scripts for Bio-ID proteomics with details on the software versions, a pipeline usage report, and protein intensity files are available from https://zenodo.org/record/8203987 All supporting data values associated with the main manuscript and supplement material, including values for all data points shown in graphs and values behind any reported means, are provided in the Supplemental Supporting Data Values file.

## Author contributions

MB (Figures 1, 2, 5, 6, and 7), CD (Figures 3 and 4), Y Li (Figures 1, 4, 6, and 7), XHZ (Figure 3), EW (Figure 4), HC (Figure 2), and ST (Figure 1) designed and performed the experiments, analyzed and interpreted the data, and wrote relevant parts of the Methods. MB, CD, Y Li, XHZ, and EW prepared figures, compiled all the Methods, and wrote the figure legends. CD, XHZ, KK, and YU analyzed Bio-ID data. DPM and KSL established relevant Bio-ID methods. XY, Y Lu, JH, and JL assisted with in vivo experiments. AK performed some of the validation experiments. EL provided the ASC-mCerulean cell line. GDC, MM, BR, and CB provided mouse strains. MC, KSL, and ZM provided intellectual input on the Rosa strain used, Bio-ID proteomics, and PPL, respectively. All authors reviewed and edited the manuscript. XL conceived the idea, initiated the project, designed and supervised the project, and wrote the manuscript. The co–first authors are listed in alphabetical order.

## Supplementary Material

Supplemental data

Supplemental table 1

Supplemental table 2

Supporting data values

## Version 1 09/12/2023

In-Press Preview

## Version 2 11/01/2023

Electronic publication

## References

[1] Martinon F (2006). Gout-associated uric acid crystals activate the NALP3 inflammasome. Nature.

[2] Strowig T (2012). Inflammasomes in health and disease. Nature.

[3] Duewell P (2010). NLRP3 inflammasomes are required for atherogenesis and activated by cholesterol crystals. Nature.

[4] Heneka MT (2013). NLRP3 is activated in Alzheimer’s disease and contributes to pathology in APP/PS1 mice. Nature.

[5] Jourdan T (2013). Activation of the Nlrp3 inflammasome in infiltrating macrophages by endocannabinoids mediates beta cell loss in type 2 diabetes. Nat Med.

[6] He Y (2016). Mechanism and Regulation of NLRP3 inflammasome activation. Trends Biochem Sci.

[7] Latz E (2013). Activation and regulation of the inflammasomes. Nat Rev Immunol.

[8] Chen J, Chen ZJ (2018). PtdIns4P on dispersed trans-Golgi network mediates NLRP3 inflammasome activation. Nature.

[9] Zhong Z (2018). New mitochondrial DNA synthesis enables NLRP3 inflammasome activation. Nature.

[10] Li X (2017). MARK4 regulates NLRP3 positioning and inflammasome activation through a microtubule-dependent mechanism. Nat Commun.

[11] Magupalli VG (2020). HDAC6 mediates an aggresome-like mechanism for NLRP3 and pyrin inflammasome activation. Science.

[12] Zitouni S (2014). Polo-like kinases: structural variations lead to multiple functions. Nat Rev Mol Cell Biol.

[13] Lens SM (2010). Shared and separate functions of polo-like kinases and aurora kinases in cancer. Nat Rev Cancer.

[14] Kishi K (2009). Functional dynamics of Polo-like kinase 1 at the centrosome. Mol Cell Biol.

[15] Blas-Rus N (2016). Aurora A drives early signalling and vesicle dynamics during T-cell activation. Nat Commun.

[16] Mahankali M (2015). A non-mitotic role for Aurora kinase A as a direct activator of cell migration upon interaction with PLD, FAK and Src. J Cell Sci.

[17] He Y (2016). NEK7 is an essential mediator of NLRP3 activation downstream of potassium efflux. Nature.

[18] Woodruff JB (2014). Pericentriolar material structure and dynamics. Philos Trans R Soc Lond B Biol Sci.

[19] Lee K, Rhee K (2011). PLK1 phosphorylation of pericentrin initiates centrosome maturation at the onset of mitosis. J Cell Biol.

[20] Kim J (2015). PLK1 regulation of PCNT cleavage ensures fidelity of centriole separation during mitotic exit. Nat Commun.

[21] Haren L (2009). Plk1-dependent recruitment of gamma-tubulin complexes to mitotic centrosomes involves multiple PCM components. PLoS One.

[22] Sdelci S (2012). Nek9 phosphorylation of NEDD1/GCP-WD contributes to Plk1 control of γ-tubulin recruitment to the mitotic centrosome. Curr Biol.

[23] Vitour D (2009). Polo-like kinase 1 (PLK1) regulates interferon (IFN) induction by MAVS. J Biol Chem.

[24] Chevrier N (2011). Systematic discovery of TLR signaling components delineates viral-sensing circuits. Cell.

[25] Hu J (2013). Polo-like kinase 1 (PLK1) is involved in toll-like receptor (TLR)-mediated TNF-α production in monocytic THP-1 cells. PLoS One.

[26] Cao Y (2018). PLK1 protects against sepsis-induced intestinal barrier dysfunction. Sci Rep.

[27] Yang XD (2020). PLK4 deubiquitination by Spata2-CYLD suppresses NEK7-mediated NLRP3 inflammasome activation at the centrosome. EMBO J.

[28] Rathbun LI (2020). PLK1- and PLK4-mediated asymmetric mitotic centrosome size and positioning in the early zebrafish embryo. Curr Biol.

[29] Hoffmann I (2022). Role of polo-like kinases Plk1 and Plk4 in the initiation of centriole duplication-impact on cancer. Cells.

[30] Sester DP (2016). Assessment of inflammasome formation by flow cytometry. Curr Protoc Immunol.

[31] Strebhardt K (2010). Multifaceted polo-like kinases: drug targets and antitargets for cancer therapy. Nat Rev Drug Discov.

[32] McInnes C (2006). Inhibitors of Polo-like kinase reveal roles in spindle-pole maintenance. Nat Chem Biol.

[33] Keppner S (2009). Identification and validation of a potent type II inhibitor of inactive polo-like kinase 1. ChemMedChem.

[34] Chen S (2012). Identification of novel, potent and selective inhibitors of Polo-like kinase 1. Bioorg Med Chem Lett.

[35] Rudolph D (2009). BI 6727, a Polo-like kinase inhibitor with improved pharmacokinetic profile and broad antitumor activity. Clin Cancer Res.

[36] Stutz A (2013). ASC speck formation as a readout for inflammasome activation. Methods Mol Biol.

[37] Sester DP (2015). A novel flow cytometric method to assess inflammasome formation. J Immunol.

[38] Clausen BE (1999). Conditional gene targeting in macrophages and granulocytes using LysMcre mice. Transgenic Res.

[39] Ventura A (2007). Restoration of p53 function leads to tumour regression in vivo. Nature.

[40] Roux KJ (2012). A promiscuous biotin ligase fusion protein identifies proximal and interacting proteins in mammalian cells. J Cell Biol.

[41] Ramanathan M (2018). RNA-protein interaction detection in living cells. Nat Methods.

[42] Firat-Karalar EN, Stearns T (2015). Probing mammalian centrosome structure using BioID proximity-dependent biotinylation. Methods Cell Biol.

[43] Burkard ME (2007). Chemical genetics reveals the requirement for Polo-like kinase 1 activity in positioning RhoA and triggering cytokinesis in human cells. Proc Natl Acad Sci U S A.

[44] Mahen R (2011). Continuous polo-like kinase 1 activity regulates diffusion to maintain centrosome self-organization during mitosis. Proc Natl Acad Sci U S A.

[45] Schnellbaecher A (2019). Vitamins in cell culture media: Stability and stabilization strategies. Biotechnol Bioeng.

[46] Samir P (2019). DDX3X acts as a live-or-die checkpoint in stressed cells by regulating NLRP3 inflammasome. Nature.

[47] Ito M (2015). Bruton’s tyrosine kinase is essential for NLRP3 inflammasome activation and contributes to ischaemic brain injury. Nat Commun.

[48] Gross O (2009). Syk kinase signalling couples to the Nlrp3 inflammasome for anti-fungal host defence. Nature.

[49] Lin YC (2015). Syk is involved in NLRP3 inflammasome-mediated caspase-1 activation through adaptor ASC phosphorylation and enhanced oligomerization. J Leukoc Biol.

[50] Lu B (2012). Novel role of PKR in inflammasome activation and HMGB1 release. Nature.

[51] Shenoy AR (2012). GBP5 promotes NLRP3 inflammasome assembly and immunity in mammals. Science.

[52] Colicino EG, Hehnly H (2018). Regulating a key mitotic regulator, polo-like kinase 1 (PLK1). Cytoskeleton (Hoboken).

[53] Archambault V, Glover DM (2009). Polo-like kinases: conservation and divergence in their functions and regulation. Nat Rev Mol Cell Biol.

[54] Archambault V (2015). Understanding the polo kinase machine. Oncogene.

[55] Khare S (2012). An NLRP7-containing inflammasome mediates recognition of microbial lipopeptides in human macrophages. Immunity.

[56] Söderberg O (2008). Characterizing proteins and their interactions in cells and tissues using the in situ proximity ligation assay. Methods.

[57] Weibrecht I (2010). Proximity ligation assays: a recent addition to the proteomics toolbox. Expert Rev Proteomics.

[58] Bauer NC (2015). Mechanisms regulating protein localization. Traffic.

[59] Wachowicz P (2016). Genetic depletion of Polo-like kinase 1 leads to embryonic lethality due to mitotic aberrancies. Bioessays.

[60] Sutterwala FS (2006). Critical role for NALP3/CIAS1/Cryopyrin in innate and adaptive immunity through its regulation of caspase-1. Immunity.

[61] Okamoto H (2021). Recombinant antithrombin attenuates acute respiratory distress syndrome in experimental endotoxemia. Am J Pathol.

[62] Maehara T (2020). Prostaglandin F_2α_ receptor antagonist attenuates LPS-induced systemic inflammatory response in mice. FASEB J.

[63] Grailer JJ (2014). Critical role for the NLRP3 inflammasome during acute lung injury. J Immunol.

[64] Liu X (2022). The Delta SARS-CoV-2 variant of concern induces distinct pathogenic patterns of respiratory disease in K18-hACE2 transgenic mice compared to the ancestral strain from Wuhan. mBio.

[65] Barretta ML (2016). Aurora-A recruitment and centrosomal maturation are regulated by a Golgi-activated pool of Src during G2. Nat Commun.

[66] Ozaki Y (2012). Poly-ADP ribosylation of Miki by tankyrase-1 promotes centrosome maturation. Mol Cell.

[67] Ramani A (2018). Plk1/Polo phosphorylates Sas-4 at the onset of mitosis for an efficient recruitment of pericentriolar material to centrosomes. Cell Rep.

[68] Kong D (2014). Centriole maturation requires regulated Plk1 activity during two consecutive cell cycles. J Cell Biol.

[69] Conduit PT (2014). The centrosome-specific phosphorylation of Cnn by Polo/Plk1 drives Cnn scaffold assembly and centrosome maturation. Dev Cell.

[70] Feldman AT, Wolfe D (2014). Tissue processing and hematoxylin and eosin staining. Methods Mol Biol.

[71] Zhou X, Moore BB (2017). Lung section staining and microscopy. Bio Protoc.

[72] Gavilan MP (2018). The dual role of the centrosome in organizing the microtubule network in interphase. EMBO Rep.

[73] Lu Y (2020). Interleukin-33 signaling controls the development of iron-recycling macrophages. Immunity.

[74] Clément M (2018). Deletion of IRF8 (interferon regulatory factor 8)-dependent dendritic cells abrogates proatherogenic adaptive immunity. Circ Res.

[75] Guarda G (2009). T cells dampen innate immune responses through inhibition of NLRP1 and NLRP3 inflammasomes. Nature.

[76] Zhang X (2008). The isolation and characterization of murine macrophages. Curr Protoc Immunol.

[77] Rajamäki K (2010). Cholesterol crystals activate the NLRP3 inflammasome in human macrophages: a novel link between cholesterol metabolism and inflammation. PLoS One.

[78] Murakami Y (2006). Induction of triggering receptor expressed on myeloid cells 1 in murine resident peritoneal macrophages by monosodium urate monohydrate crystals. Arthritis Rheum.

[79] Li X (2004). FRS2-dependent SRC activation is required for fibroblast growth factor receptor-induced phosphorylation of Sprouty and suppression of ERK activity. J Cell Sci.

[80] Schopp IM (2017). Split-BioID a conditional proteomics approach to monitor the composition of spatiotemporally defined protein complexes. Nat Commun.

[81] Seidi A (2018). Elucidating the mitochondrial proteome of *Toxoplasma gondii* reveals the presence of a divergent cytochrome *c* oxidase. Elife.

[82] Shi H (2016). NLRP3 activation and mitosis are mutually exclusive events coordinated by NEK7, a new inflammasome component. Nat Immunol.

