# Supplementary material for: PLK1 inhibition dampens NLRP3 inflammasome–elicited response in inflammatory disease models
Source: J Clin Invest. 2023 Nov 1;133(21):e162129. doi: 10.1172/JCI162129 (PMC10617773; doi:10.1172/JCI162129)

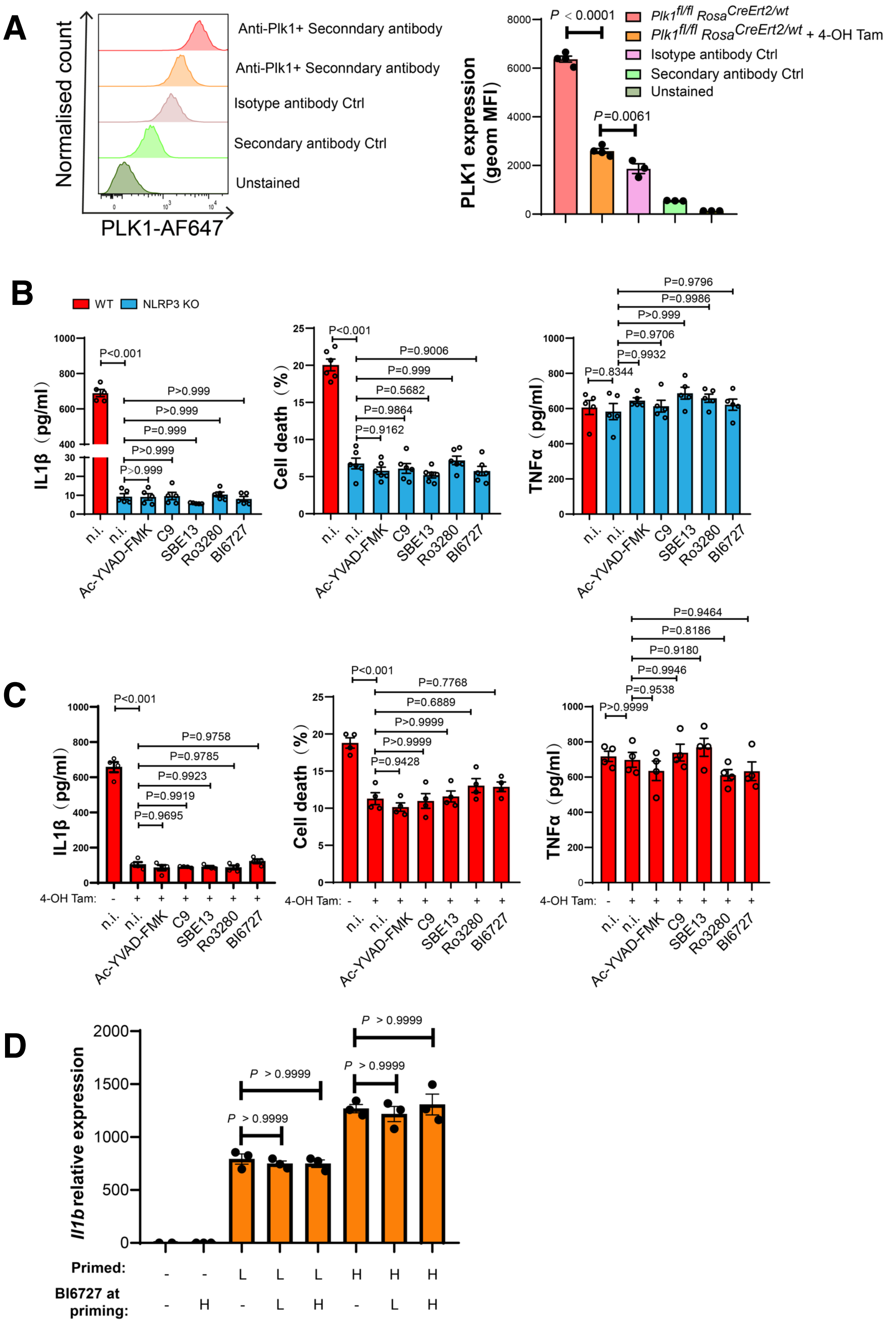

Figure S1: Validation of PLK1 detection and examination of *Il1b* expression and IL1β production under PLK1 inhibition.

(A) *Rosa*$^{CreErt2/wt}$*Plk1*$^{fl/fl}$ BMDMs were treated with 4-OH Tamoxifen (0.002mg/mL 4-OH Tam, 24 hours), and cells treated without 4-OH Tam were used as controls. Cells were then stained with PLK1 for flow cytometry analysis. Isotype antibody control (Ctrl) and secondary antibody control were used. n= 4, 4, 3, 3 and 3, in order from the left column to the right. **(B)** Primed (100ng/mL LPS) NLRP3 knock-out (KO) BMDMs were activated (5mM ATP, 30 minutes), with or without selective PLK1 inhibitors (Cyclapolin9 (C9) 3μM; SBE13 10nM; Ro3280 50nM; BI6727 0.8nM) at the activation stage. The supernatants were collected to measure IL1β concentration (n=5/group), cell death (n=6/group) and TNFα concentration (n=5/group). Treated samples from wide-type (WT) BMDMs without selective PLK1 inhibitors were used as control. **(C)** *Rosa*$^{CreErt2/wt}$*Plk1*$^{fl/fl}$ BMDMs were treated with 0.002mg/mL 4-OH Tam for 24 hours before and during priming (100ng/mL LPS, 5 hours), and then cells were activated (5mM ATP, 30 minutes) with or without selective PLK1 inhibitors (3μM Cyclapolin9 (C9); 10nM SBE13; 50nM Ro3280; 0.8nM BI6727). The supernatants were collected to measure IL1β concentration, cell death and TNFα concentration (n=4/group). Cells without 4-OH Tam treatment were used as control. **(D)** Murine BMDMs primed with low (L, 100ng/mL) or high (H, 500ng/mL) dose of LPS, were co-treated with the PLK1 inhibitor BI6727 at low (L, 0.4nM) or high (H, 0.8nM) dose. *Il1b* relative expression was quantified by qPCR (n= 2, 3, 3, 3, 3, 3, 3 and 3, in order from the left column to the right). Results are representative of 2 (**A-C**) or 3 (**D**) independent experiments. One-way ANOVA with Tukey post hoc test (**A-C**) and two-way ANOVA with Sidak post hoc test (**D**) were used for statistical analysis. All data are mean±SEM.

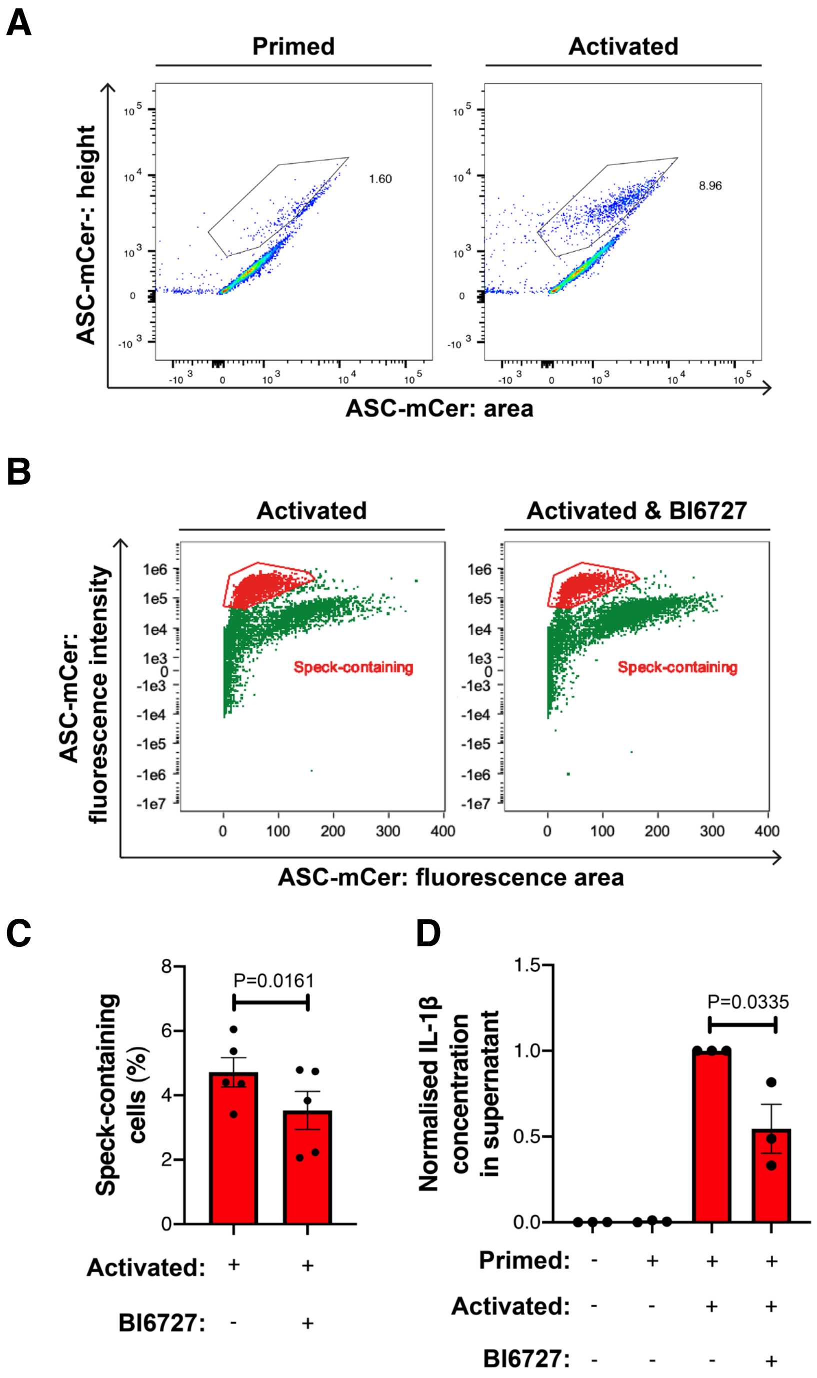

Figure S2: Validation of PLK1 effect on speck formation.

(**A**) mCerulean fluorescence area and height were measured on a flow cytometer to confirm speck detection, in immortalized BMDMs (iBMDMs) expressing ASC-mCerulean. These cells were primed (100ng/mL LPS, 5 hours) and activated (5μM nigericin, 2 hours). Cells without activation (primed) were used as controls. (**B, C, D**) iBMDMs expressing ASC-mCerulean were primed (100ng/mL LPS, 5 hours), and then activated (2.5μM nigericin, 30 minutes) under PLK1 inhibition (0.8nM BI6727). ASC specks were identified and quantified by imaging flow cytometry. Representative scatter plots **(B)** and quantification (**C**). Supernatants were collected for IL1β quantification by ELISA (**D**). Cells without PLK1 inhibition were used as control. Results are representative of 3 independent replicates **(A, B),** or represent pooled data from 5 independent replicates (**C**). In (**D**), results represent pooled data from 3 independent biological replicates (each replicate comprises all 4 experimental conditions), and normalized IL1β concentration was used to account for differences in plated cell numbers across different biological replicates. Paired t-test was used for statistical analysis (**C, D**). All data are mean ± SEM.

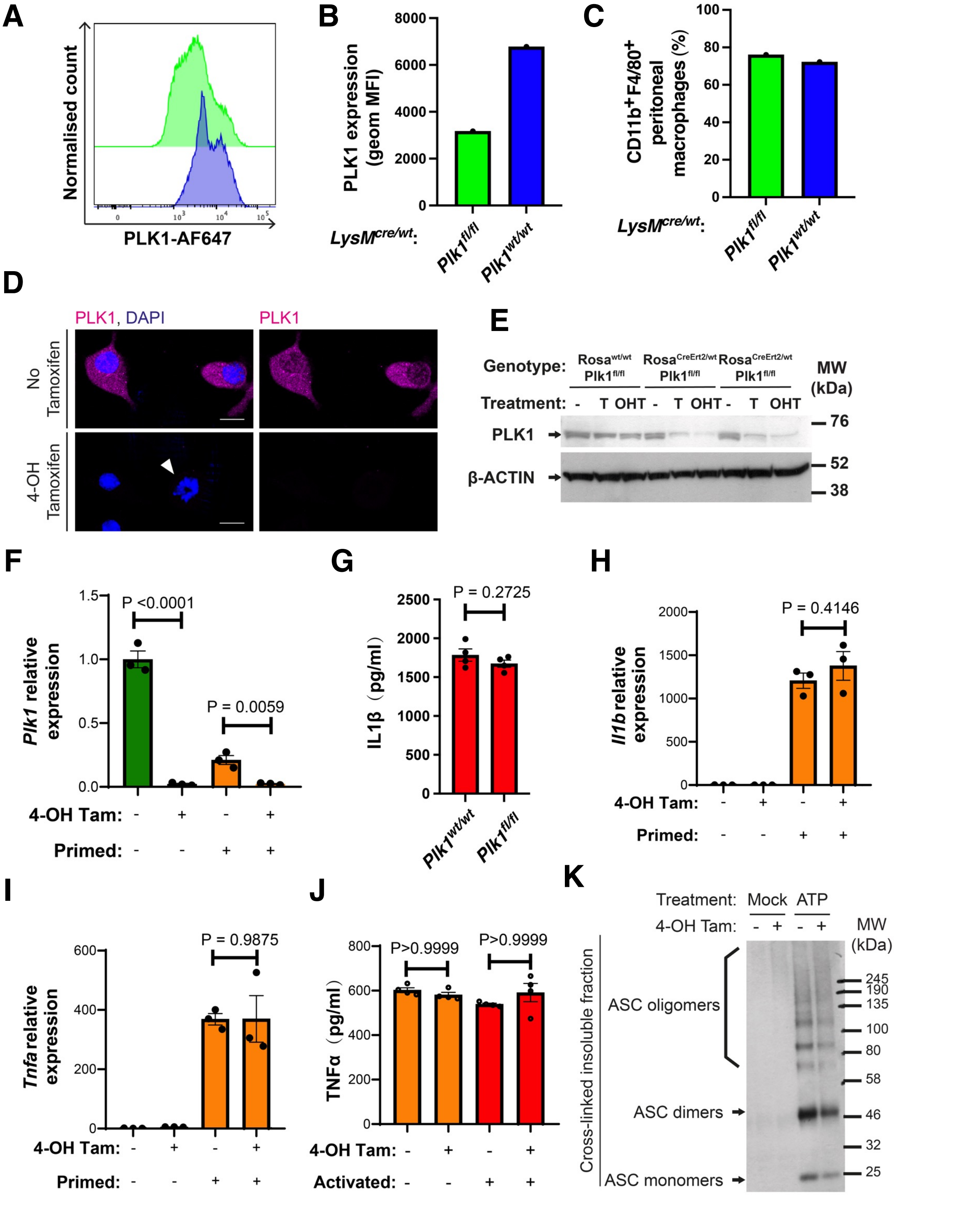

Figure S3: Validation of PLK1 genetic depletion and its effect on NLRP3 inflammasome activation.
(A-C) Peritoneal macrophages were elicited with 1mL of 3% thioglycolate (i.p.) for 4 days and isolated by AutoMACS separation. Cells were then analyzed on a flow cytometer to quantify PLK1 levels (**A, B**), and to evaluate purity **(C)**. Control, *LysM*$^{cre/wt}$ *Plk1*$^{wt/wt}$; deletion, *LysM*$^{cre/wt}$ *Plk1*$^{fl/fl}$. **(D)** *Rosa*$^{CreErt2/wt}$*Plk1*$^{fl/fl}$ BMDMs were treated with 4-OH Tamoxifen (0.002mg/mL 4-OH Tam, 24 hours), and then stained for PLK1 to assess protein levels. Arrowhead indicates a monopolar spindle of an arresting cell in mitosis, a phenotype of effective PLK1 inhibition. Scale bar = 10μm. **(E)** *Rosa*$^{CreErt2/wt}$*Plk1*$^{fl/fl}$ and *Rosa*$^{wt/wt}$ *Plk1*$^{fl/fl}$ BMDMs were treated with 0.002mg/mL tamoxifen (T) or 0.002mg/mL 4-OH tamoxifen (OHT) for 24 hours, and cell lysates were then analyzed by western blot. **(F, H, I)** *Rosa*$^{CreErt2/wt}$*Plk1*$^{fl/fl}$ BMDMs were treated with 0.002mg/mL 4-OH Tam for 24 hours before and during priming (100ng/mL LPS, 5 hours) to deplete PLK1. *Plk1* **(F)**, *Il1b* **(H)** and *Tnfa* **(I)** mRNA relative expression was quantified by qPCR. n=3/group. **(G)** BMDMs derived from *Plk1*$^{fl/fl}$, and *Plk1*$^{wt/wt}$ mice primed with LPS (100ng/mL, 5 hours) and activated with ATP (5mM, 30 minutes). Supernatants were collected for IL1β quantification by ELISA. n=4/group. **(J)** Supernatants from *Rosa*$^{CreErt2/wt}$ *Plk1*$^{fl/fl}$ BMDMs treated as in **(F)** were collected for TNFα quantification by ELISA. n=4/group. **(K)** *Rosa*$^{CreErt2/wt}$*Plk1*$^{fl/fl}$ BMDMs were treated with 0.002mg/mL 4-OH Tam for 24 hours before and during priming (100ng/mL LPS, 5 hours) and then activated (5mM ATP, 30 minutes). Cross-linked insoluble protein fractions from those cells were analyzed by western blot to detect ASC oligomer formation. Results are representative of 3 **(A, B, C)** or 2 **(F, H, I)** independent experiments. Two-way ANOVA with Sidak post hoc test (**F, H, I, J, K**), and unpaired t test (**G**) were used for statistical analysis. All data are mean±SEM.

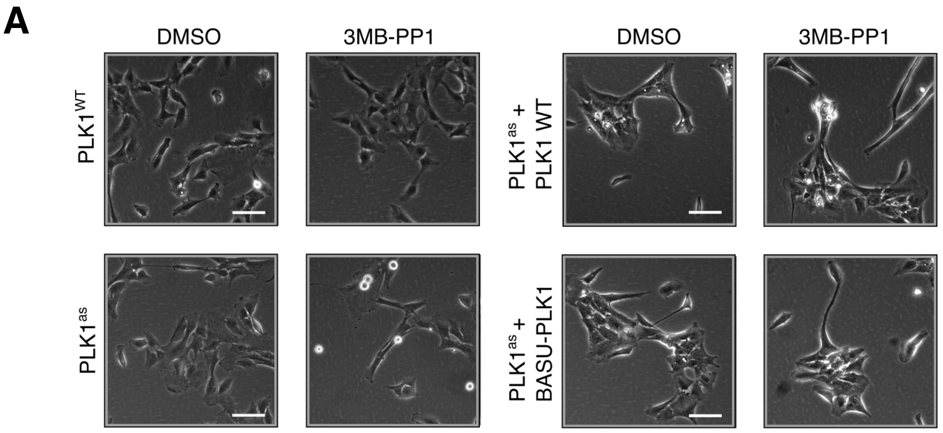

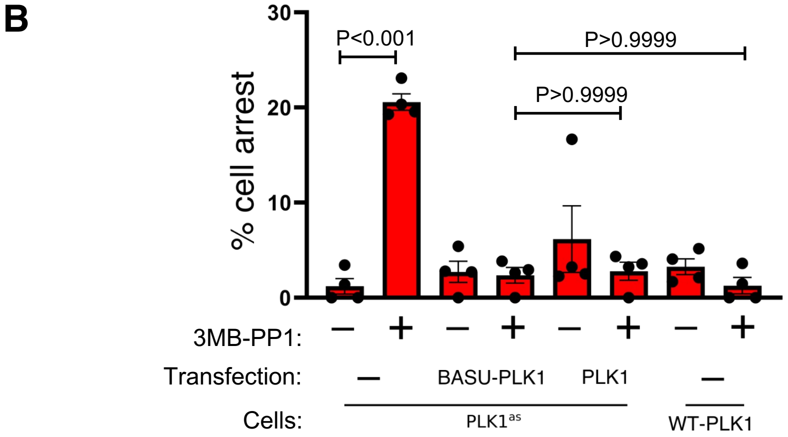

Figure S4. Validation of BASU-tagged PLK1.

tRPE PLK1as cells were transfected with wild-type PLK1 or BASU-tagged PLK1 as indicated. tRPE PLK1as cells were incubated with 3MB-PP1 as indicated to inhibit PLK1, and wild-type tRPE cells were used as controls. (**A**) Representative views used to count round cells in cell cycle arrest. Scale bar= 50µm. (**B**) Quantitative data of cells in cell cycle arrest (%). n=4/group. Two-way ANOVA with Sidak post hoc test was used. All data are mean ± SEM.

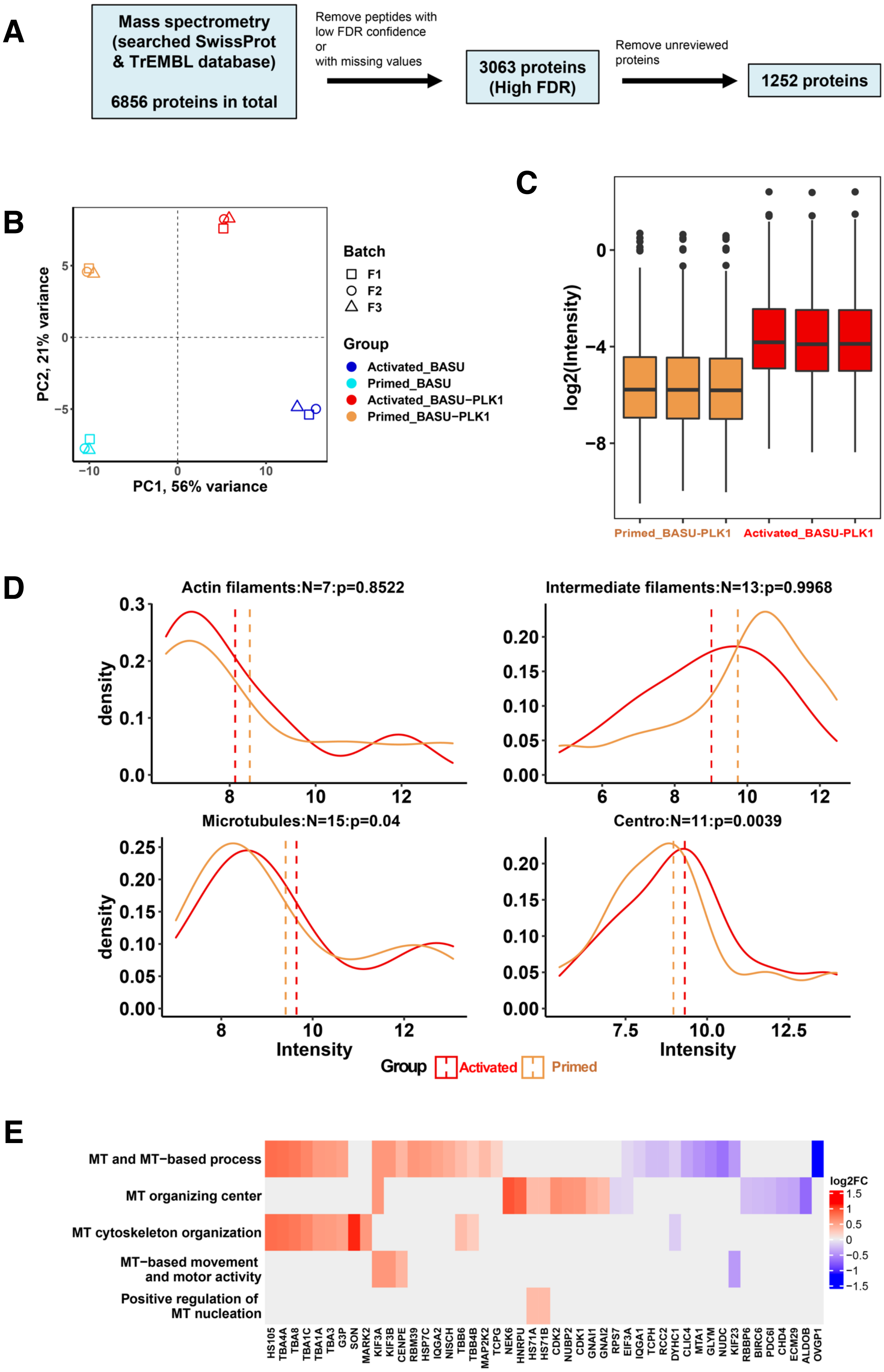

Figure S5. Bio-ID identified increased interactome with PLK1 after NLRP3 inflammasome activation.
(**A**) The analyzed interacting proteins which have passed the quality control are shown in the flow chart. (**B**) The interactome was analyzed under primed condition (primed), or after NLRP3 inflammasome activation (activated). PCA (Principal Components Analysis) shows the grouped replicates under each condition (BASU only under Primed or Activated condition; BASU-PLK1 under Primed or Activated condition). (**C**) Boxplot of normalized intensities of interacting proteins with PLK1 under primed condition or activated condition upon normalization using central median then PLK1 in each batch. (**D**) Density plot shows intensity shift of interacting proteins with PLK1 under primed or activated conditions at four subcellular cytoskeletal localization. Paired t-test for each location group is performed. (**E**) Heatmap plot shows the up (red shades) /down (blue shades)-regulated interacting proteins with PLK1 in microtubule-related pathways after NLRP3 inflammasome activation.

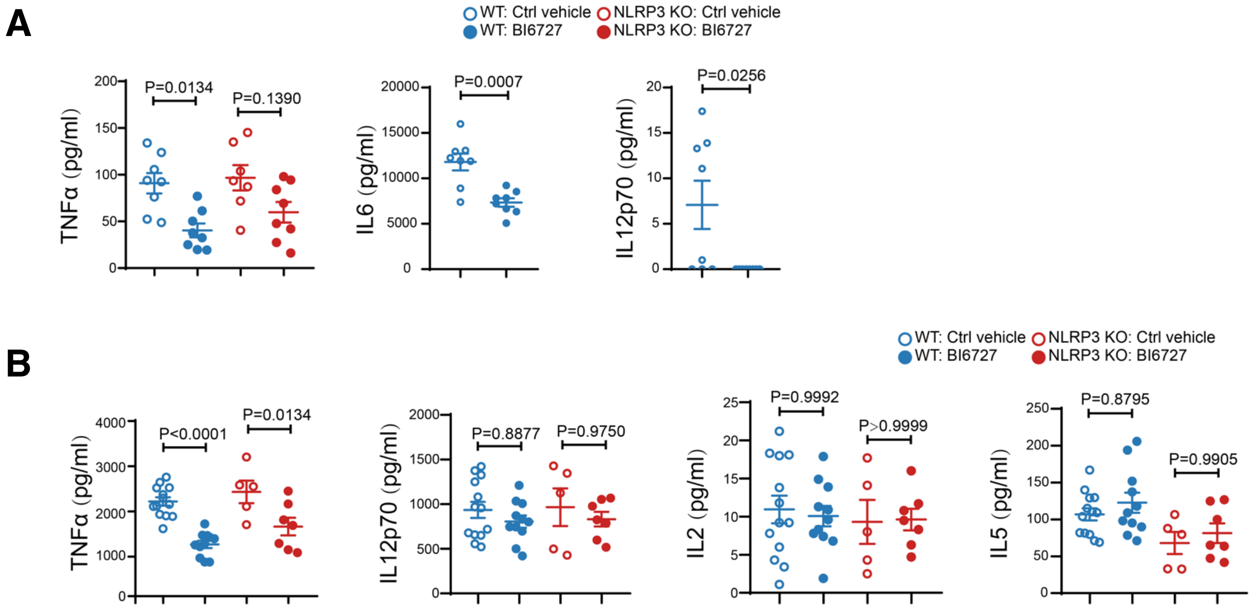

Figure S6. The effect of PLK1 inhibition on cytokines in LPS-induced endotoxemia model.
C57BL/6 wild-type (WT) and NLRP3 knock-out (KO) mice were treated with BI6727 (5mg/kg, i.p.) or control vehicle, followed by LPS administration (20mg/kg, i.p.). Samples for cytokine measurement were collected at 3 hours after LPS administration. (**A**) Peritoneal lavage fluid samples were collected, and multiple cytokine levels were measured. IL6 and IL12 were undetectable in NLRP3 KO samples WT: Ctrl vehicle, n=8; WT: BI6727, n=8; NLRP3 KO: Ctrl vehicle, n=7; NLRP3 KO: BI6727, n=8. (**B**) Serum samples were collected, and multiple cytokine levels were measured. WT: Ctrl vehicle, n= 13; WT: BI6727, n=11; NLRP3 KO: Ctrl vehicle, n=5; NLRP3 KO: BI6727 group, n=7. Unpaired t-test was used to compare two groups, and two-way ANOVA with Sidak post hoc test was used to compare multiple groups for statistical analysis. Non-parametric test was used to analyze the data of IL12p70 level in **(A)**. All data are mean ± SEM.

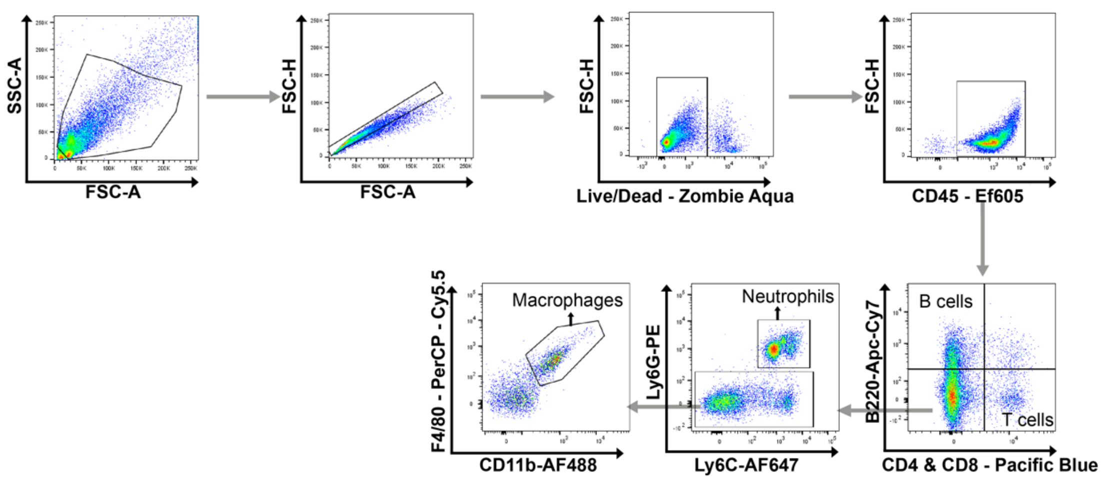

Figure S7. The gating strategy when examining the effect of PLK1 inhibition on immune cells in MSU-induced peritonitis model.

C57BL/6 wild-type (WT) and NLRP3 knock-out (KO) mice were treated with BI6727 (1mg/kg, i.v.) or control vehicle, followed by MSU (0.5mg/mouse, i.p.) for 6 hours. The cells were collected from the peritoneal cavity and analyzed on a flow cytometer. The gating strategy was shown.

Full unedited gel for all Figures

Figure 2D

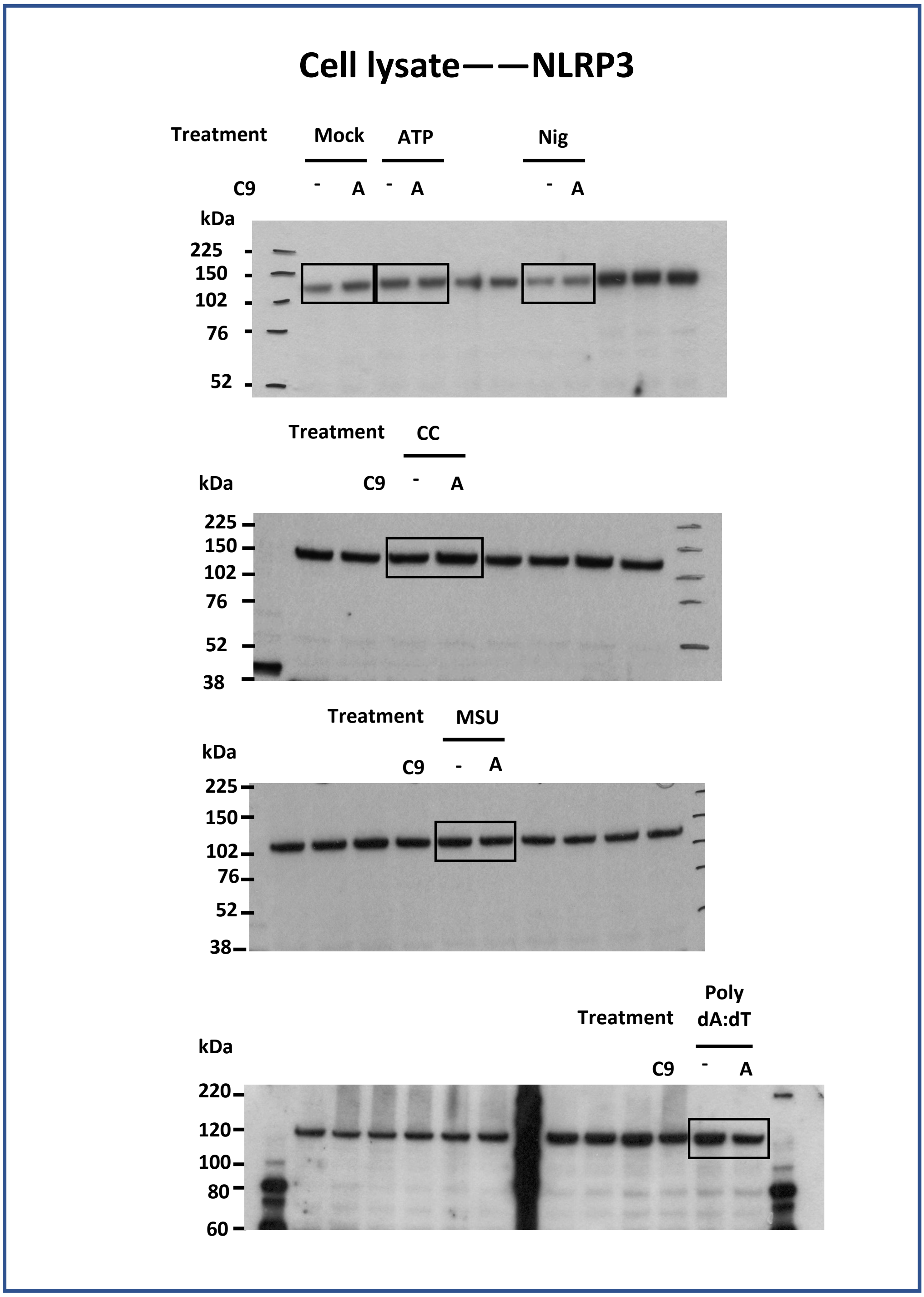

Cell lysate——NLRP3
Treatment
Mock
ATP
Nig
C9
-
A
-
A
-
A
kDa
225
150
102
76
52
Treatment
CC
kDa
C9
-
A
225
150
102
76
52
38
Treatment
MSU
kDa
C9
-
A
225
150
102
76
52
38
Poly
dA:dT
Treatment
kDa
C9
-
A
220
120
100
80
60

Figure 2D

Cell lysate——Pro-Casp1

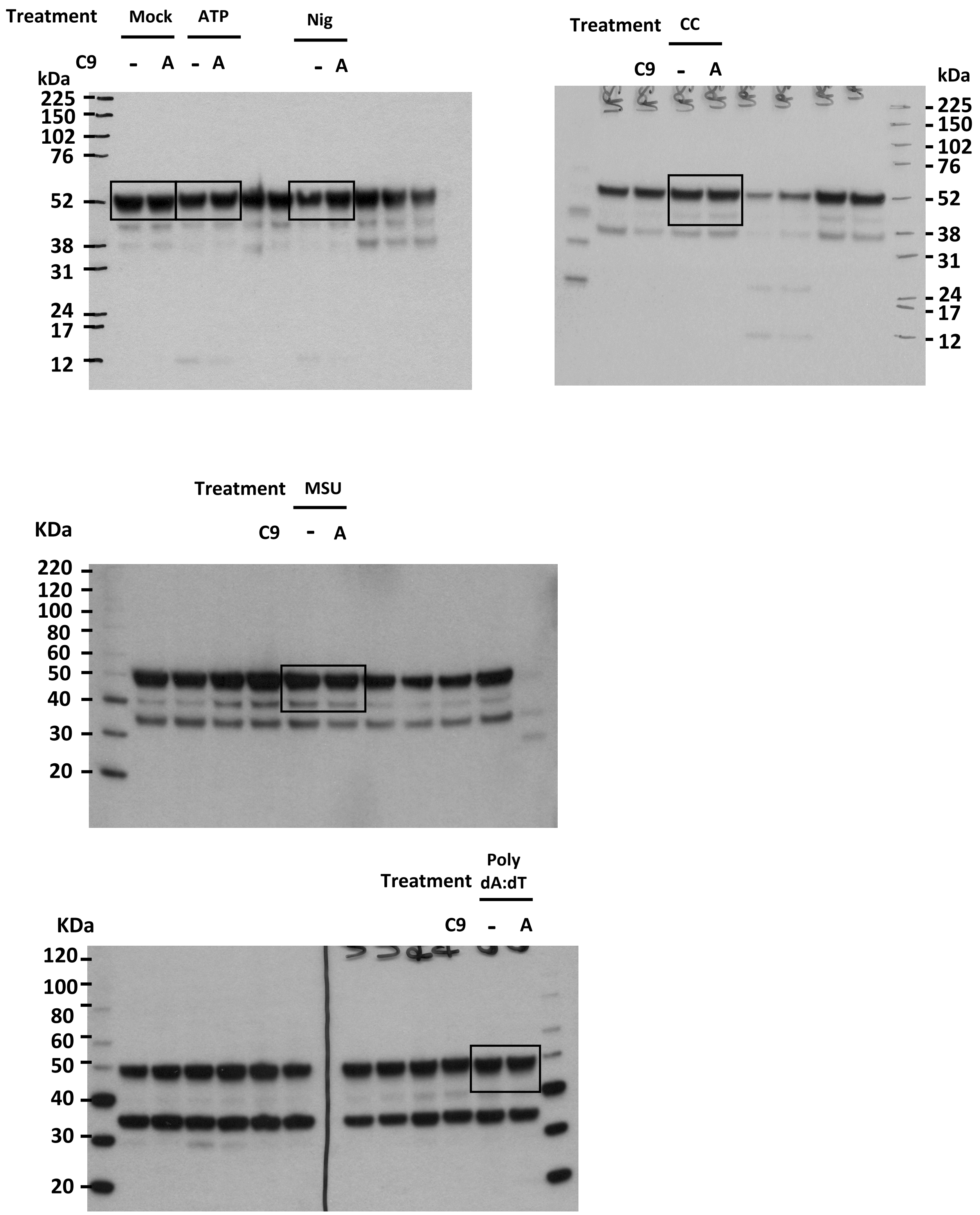

Figure 2D

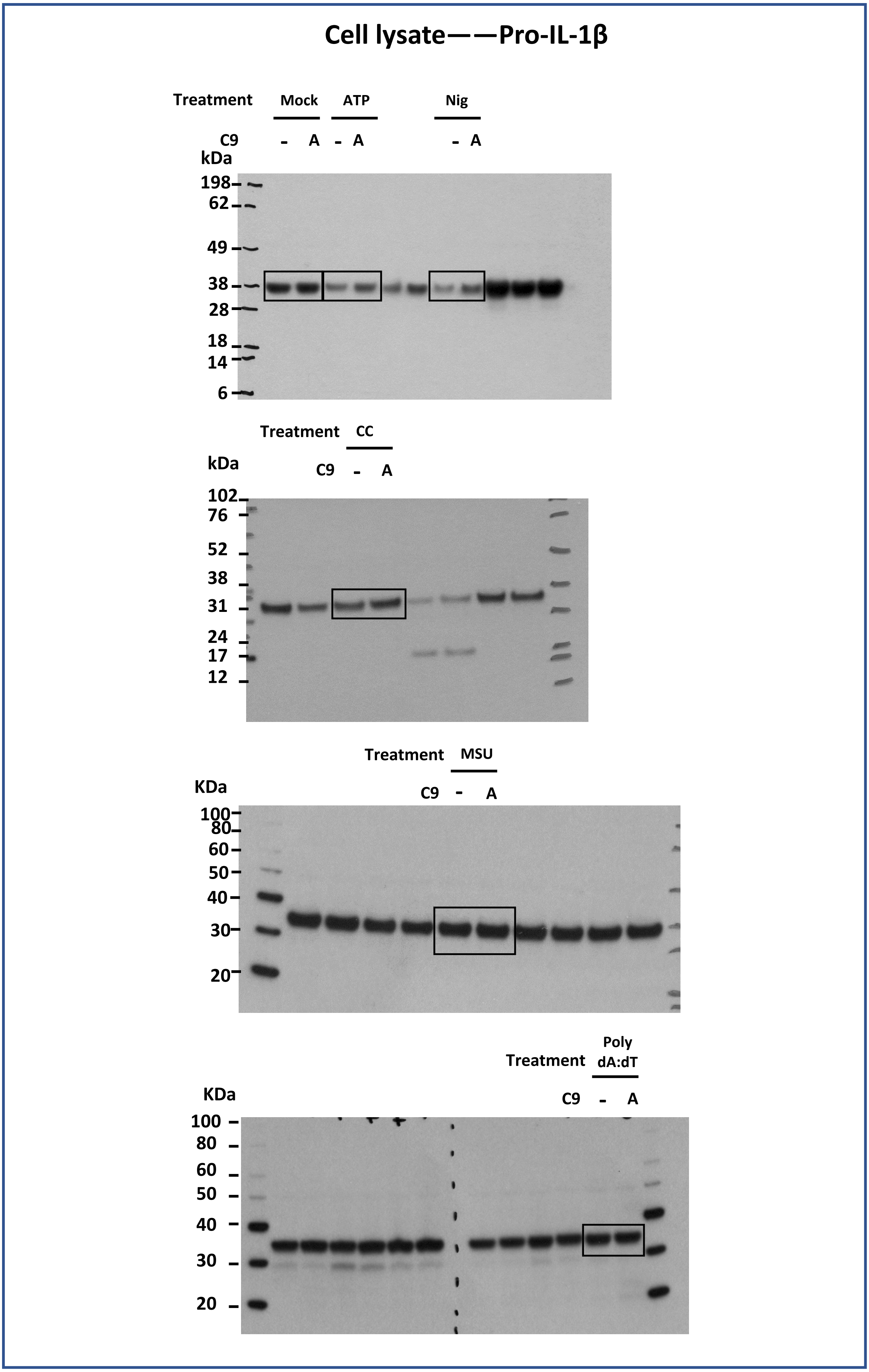

Cell lysate——Pro-IL-1β
Treatment
Mock
ATP
Nig
C9
- A - A - A
kDa
198
62
49
38
28
18
14
6
Treatment
CC
kDa
C9
- A
102
76
52
38
31
24
17
12
Treatment
MSU
KDa
C9
- A
100
80
60
50
40
30
20
Treatment
Poly dA:dT
KDa
C9
- A
100
80
60
50
40
30
20

Figure 2D

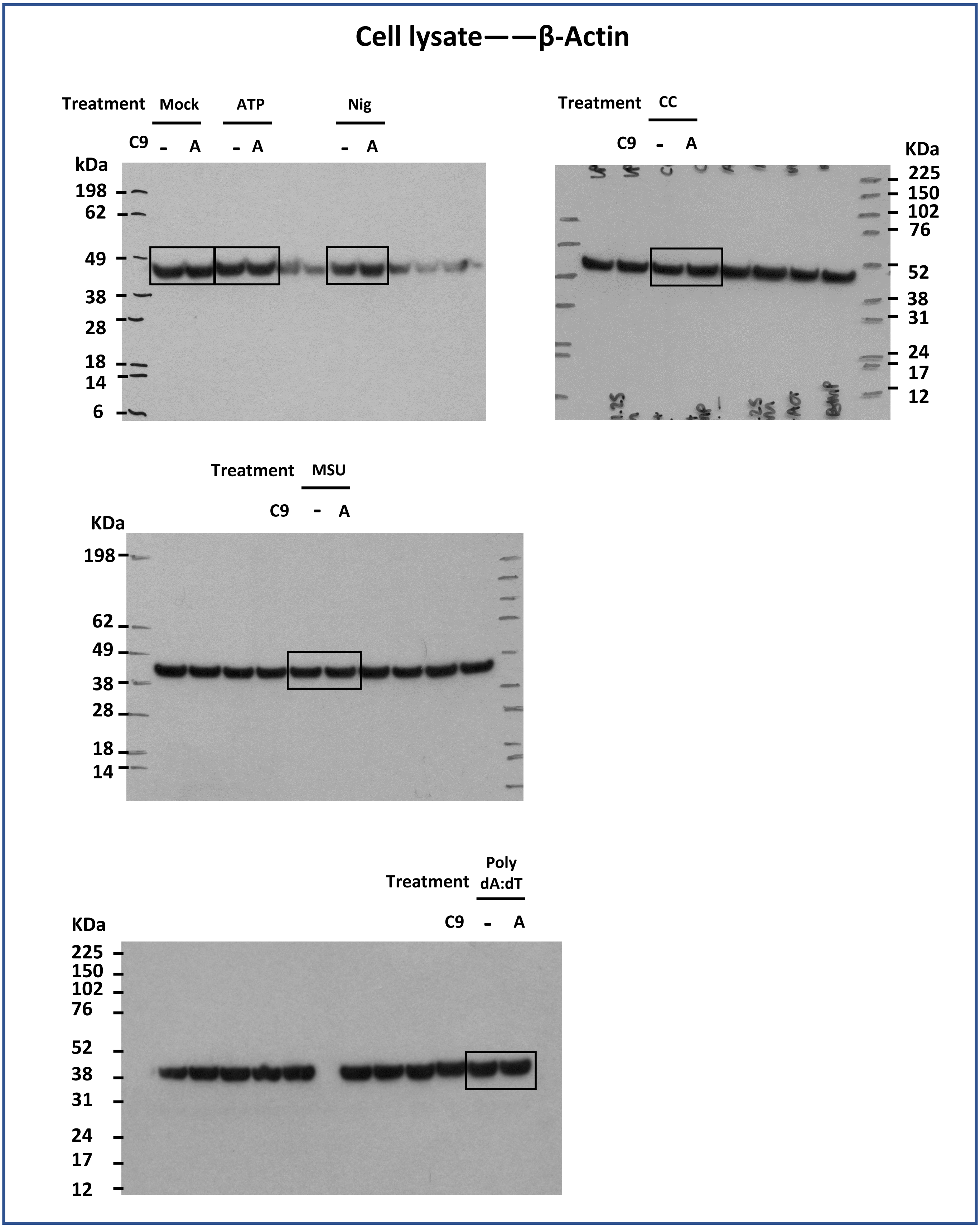
Cell lysate——β-Actin
Treatment Mock ATP Nig
C9 - A - A - A
kDa
198
62
49
38
28
18
14
6
Treatment CC
C9 - A
KDa
225
150
102
76
52
38
31
24
17
12
Treatment MSU
C9 - A
KDa
198
62
49
38
28
18
14
Treatment Poly dA:dT
C9 - A
KDa
225
150
102
76
52
38
31
24
17
12

Figure 2D

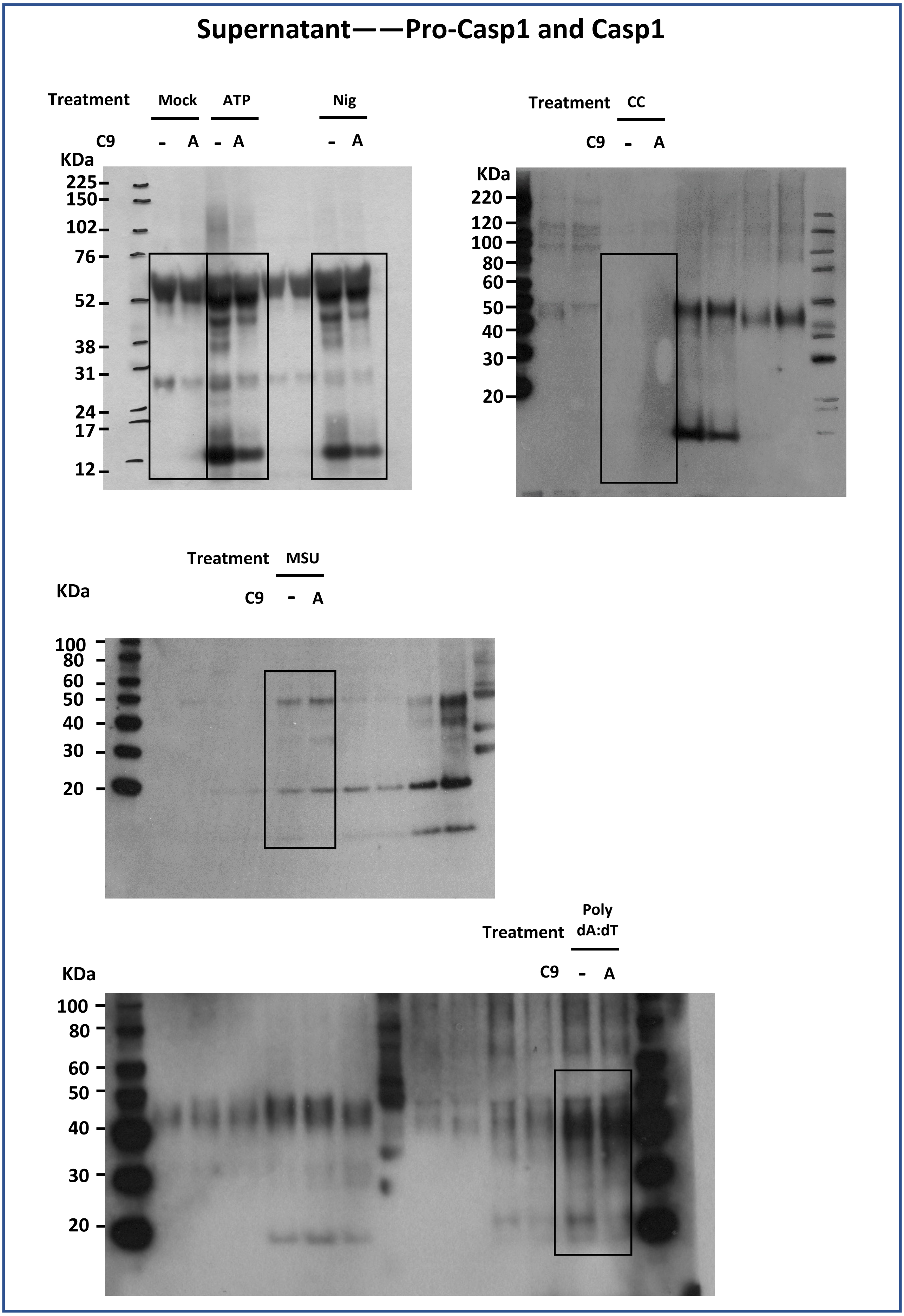

Supernatant——Pro-Casp1 and Casp1
Treatment
Mock
ATP
Nig
C9
- A - A - A
KDa
225
150
102
76
52
38
31
24
17
12
Treatment
CC
C9
- A
KDa
220
120
100
80
60
50
40
30
20
Treatment
MSU
C9
- A
KDa
100
80
60
50
40
30
20
Treatment
Poly dA:dT
C9
- A
KDa
100
80
60
50
40
30
20

Figure 2D

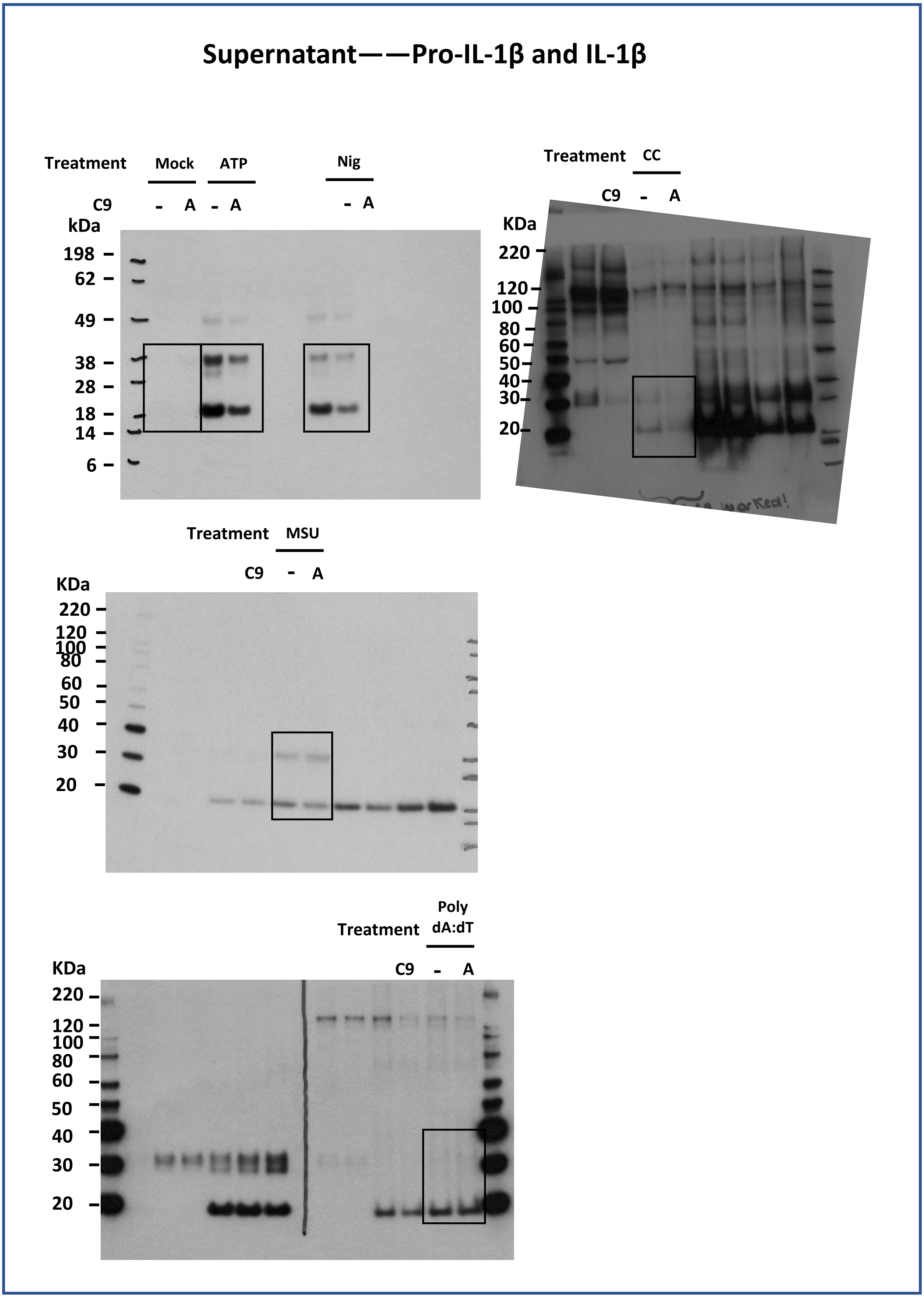
Supernatant——Pro-IL-1β and IL-1β
Treatment
Mock
ATP
Nig
C9
- A - A
- A
kDa
198
62
49
38
28
18
14
6
Treatment
CC
C9
- A
KDa
220
120
100
80
60
50
40
30
20
Treatment
MSU
C9
- A
KDa
220
120
100
80
60
50
40
30
20
Treatment
Poly dA:dT
C9
- A
KDa
220
120
100
80
60
50
40
30
20

Figure 2G

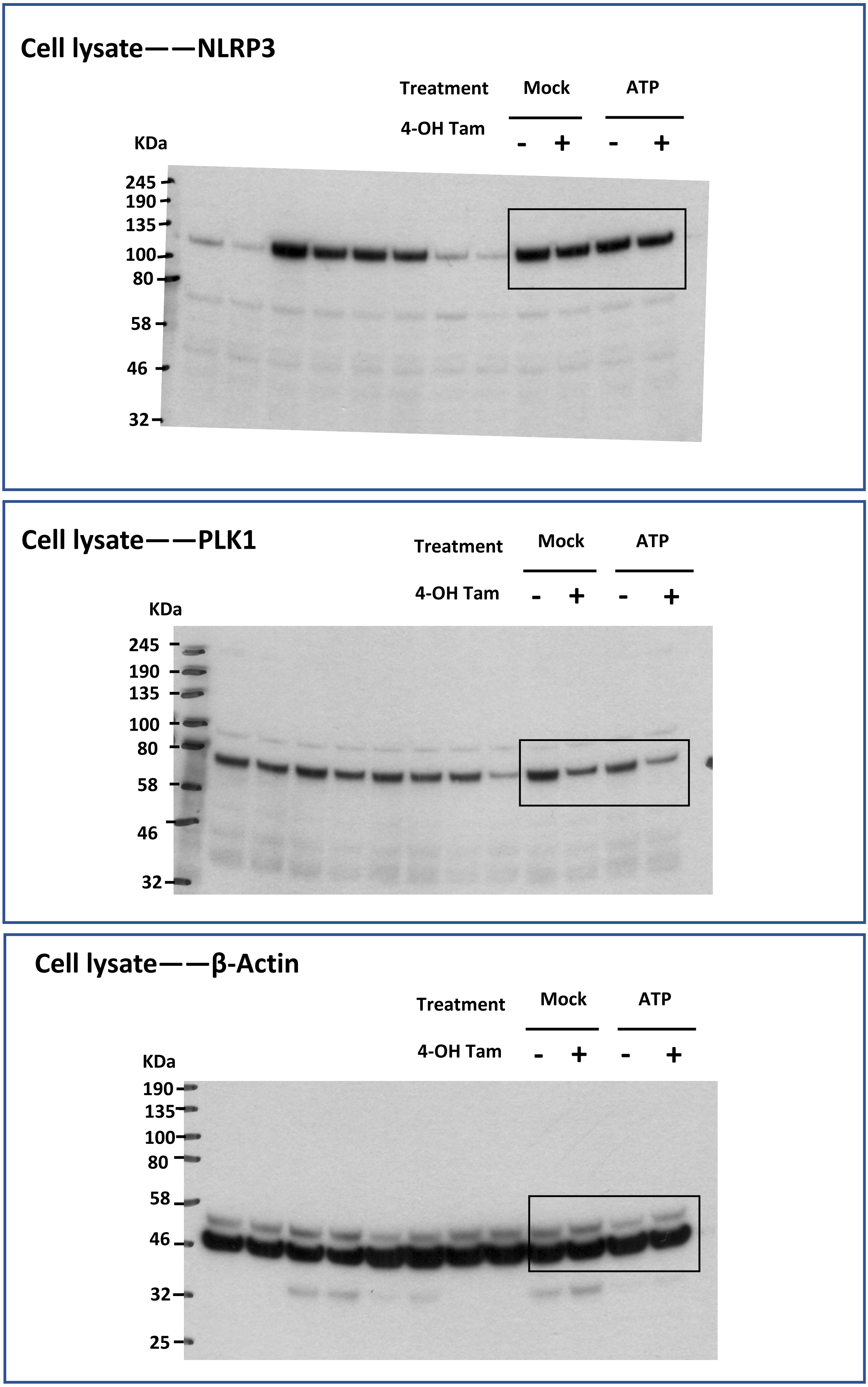
Cell lysate——NLRP3
Treatment
Mock
ATP
4-OH Tam
- + - +
KDa
245
190
135
100
80
58
46
32
Cell lysate——PLK1
Treatment
Mock
ATP
4-OH Tam
- + - +
KDa
245
190
135
100
80
58
46
32
Cell lysate——β-Actin
Treatment
Mock
ATP
4-OH Tam
- + - +
KDa
190
135
100
80
58
46
32
25

Figure 2G

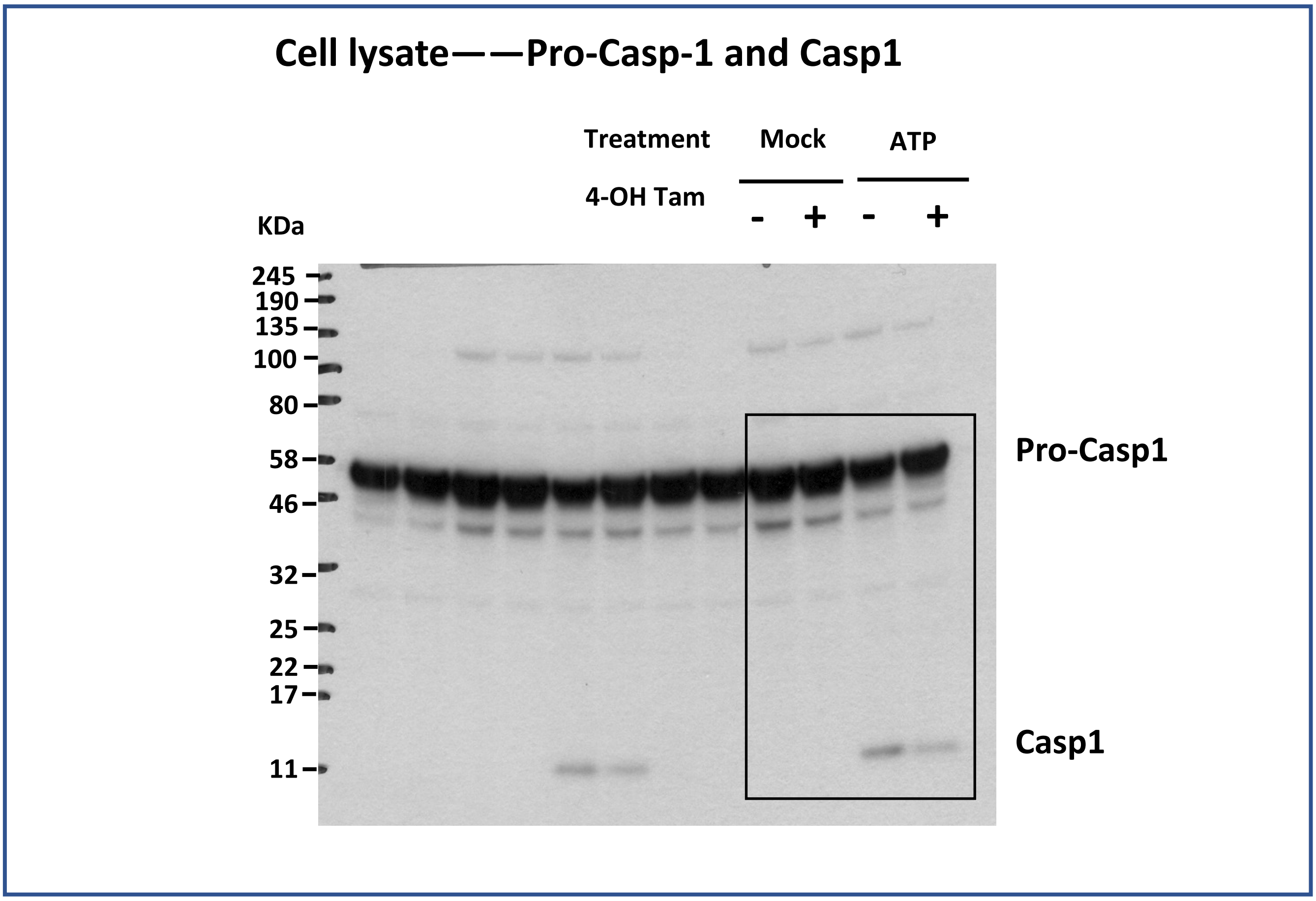
Cell lysate——Pro-Casp-1 and Casp1
Treatment
Mock
ATP
4-OH Tam
- + - +
KDa
245
190
135
100
80
58
46
32
25
22
17
11
Pro-Casp1
Casp1

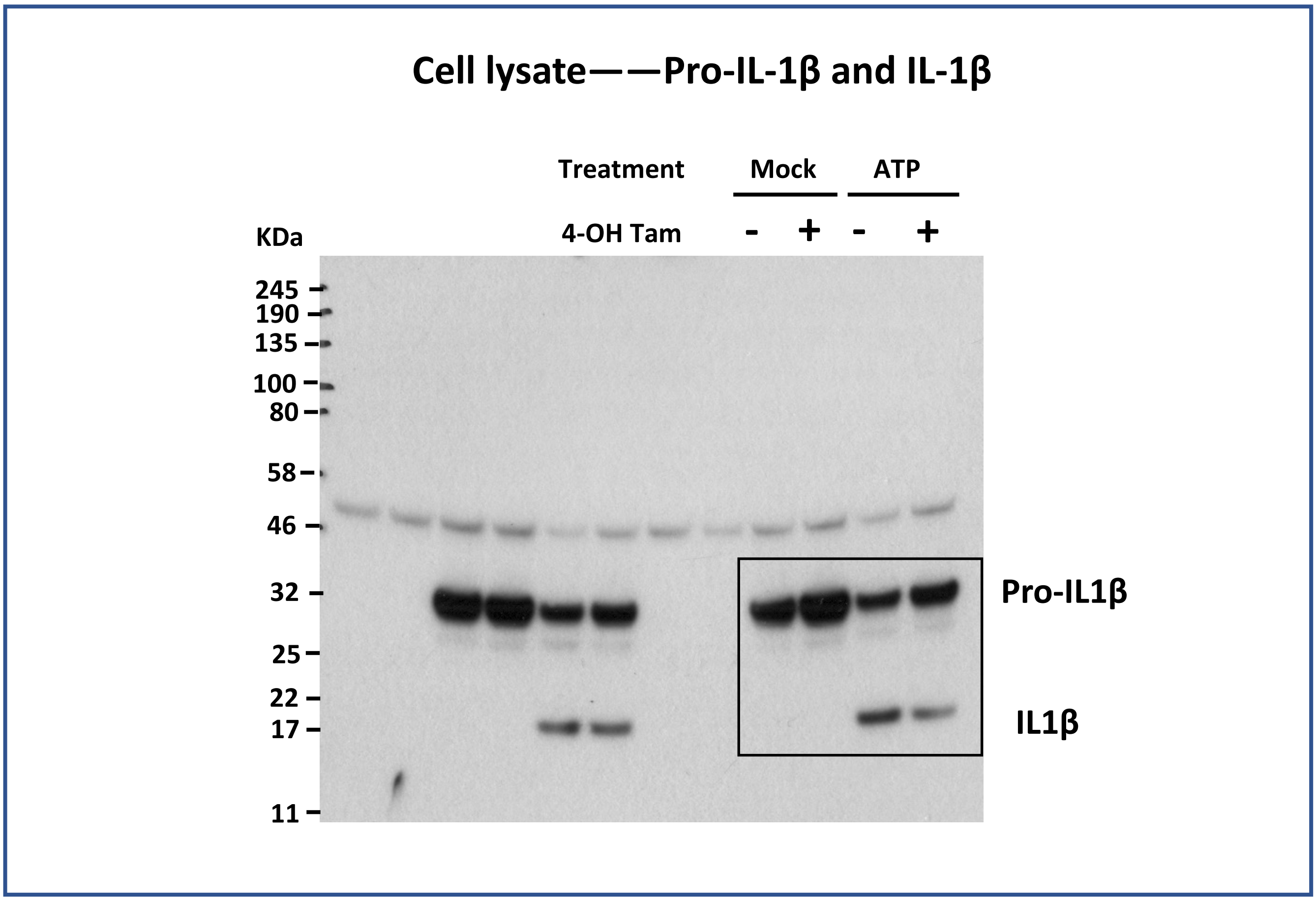
Cell lysate——Pro-IL-1β and IL-1β
Treatment
Mock
ATP
4-OH Tam
- + - +
KDa
245
190
135
100
80
58
46
32
25
22
17
11
Pro-IL1β
IL1β

Figure 2G

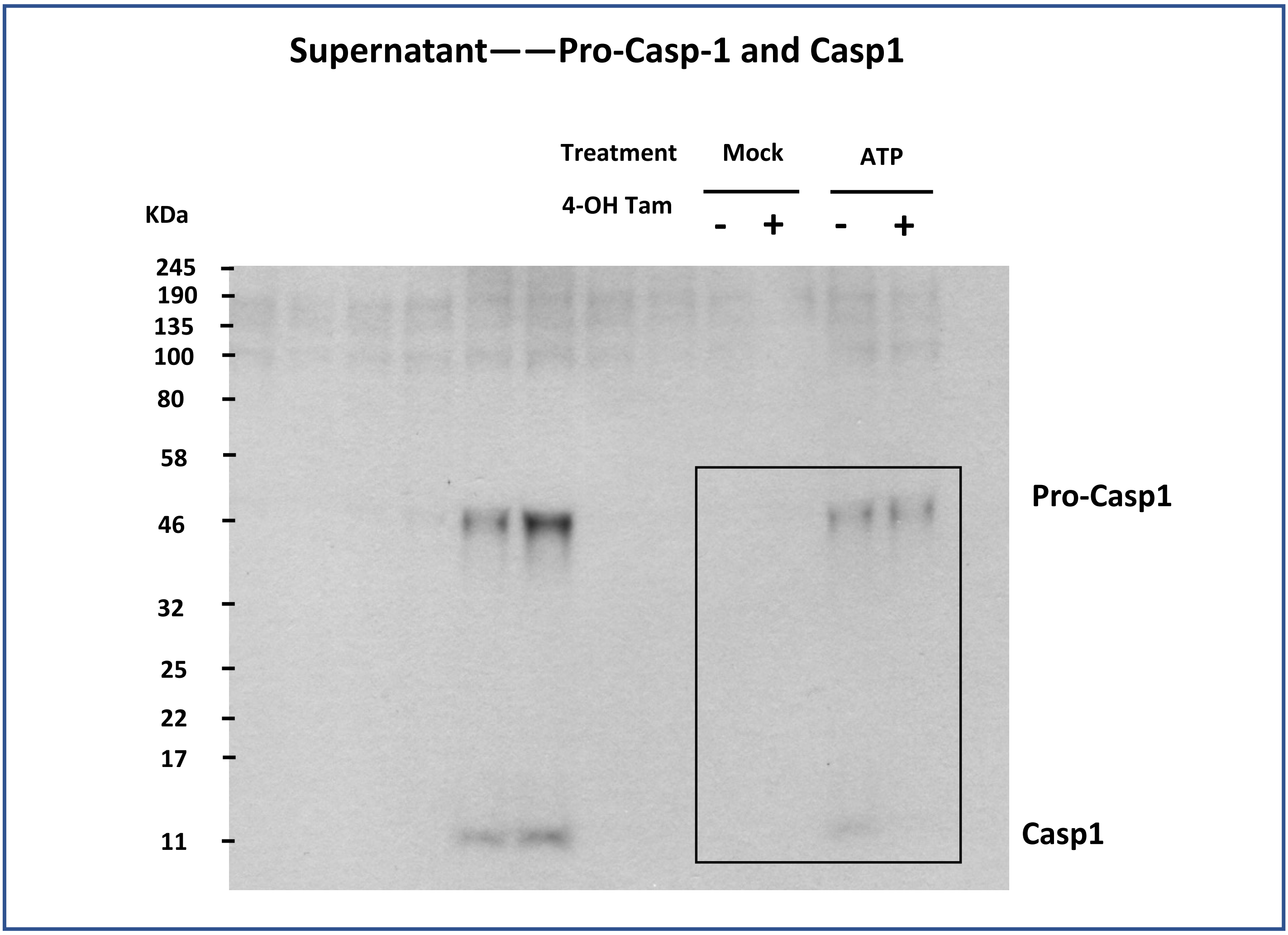

Supernatant——Pro-Casp-1 and Casp1
Treatment
Mock
ATP
4-OH Tam
- + - +
KDa
245
190
135
100
80
58
46
32
25
22
17
11
Pro-Casp1
Casp1

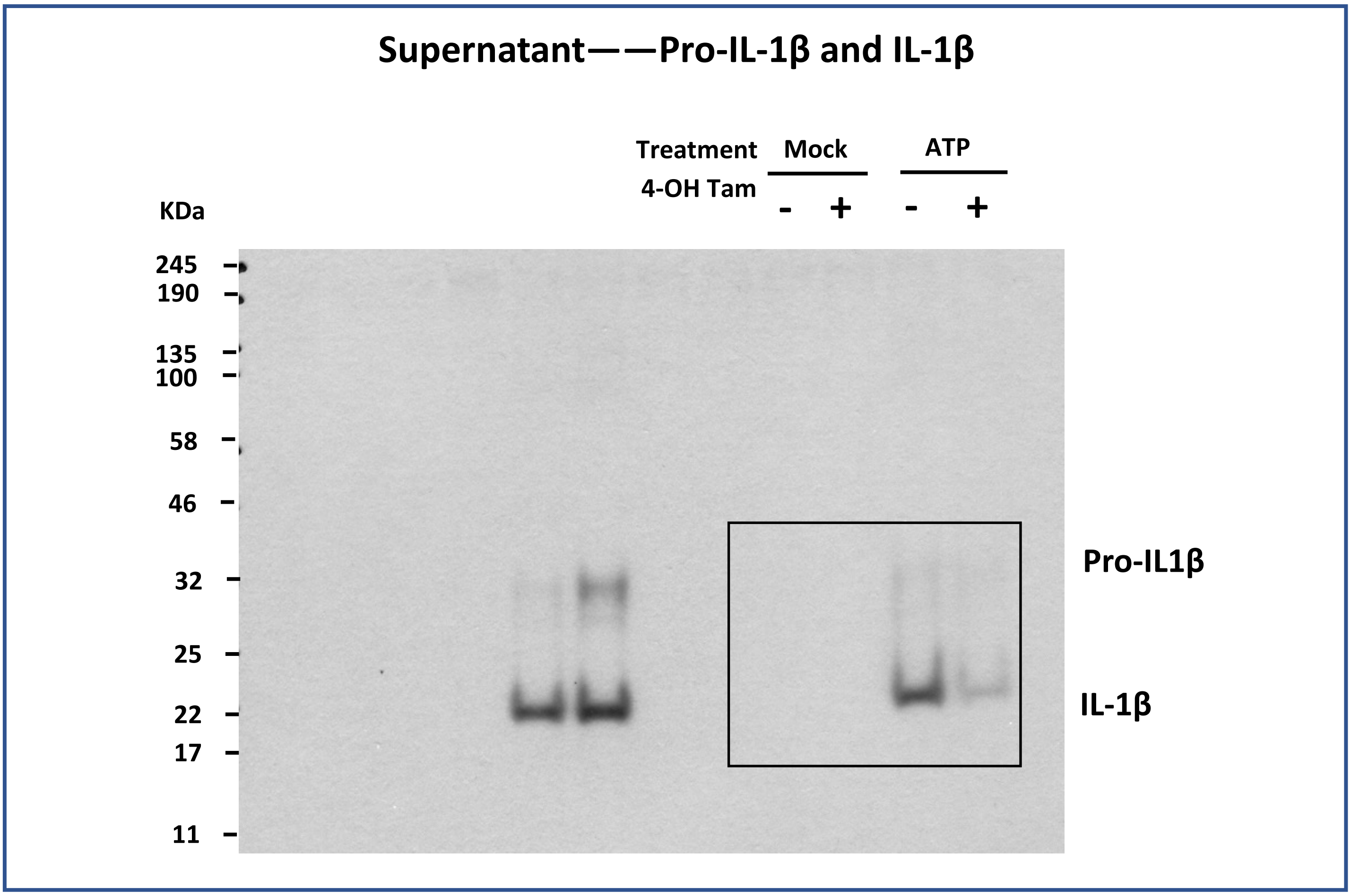

Supernatant——Pro-IL-1β and IL-1β
Treatment
Mock
ATP
4-OH Tam
- + - +
KDa
245
190
135
100
58
46
32
25
22
17
11
Pro-IL1β
IL-1β

Figure 3C

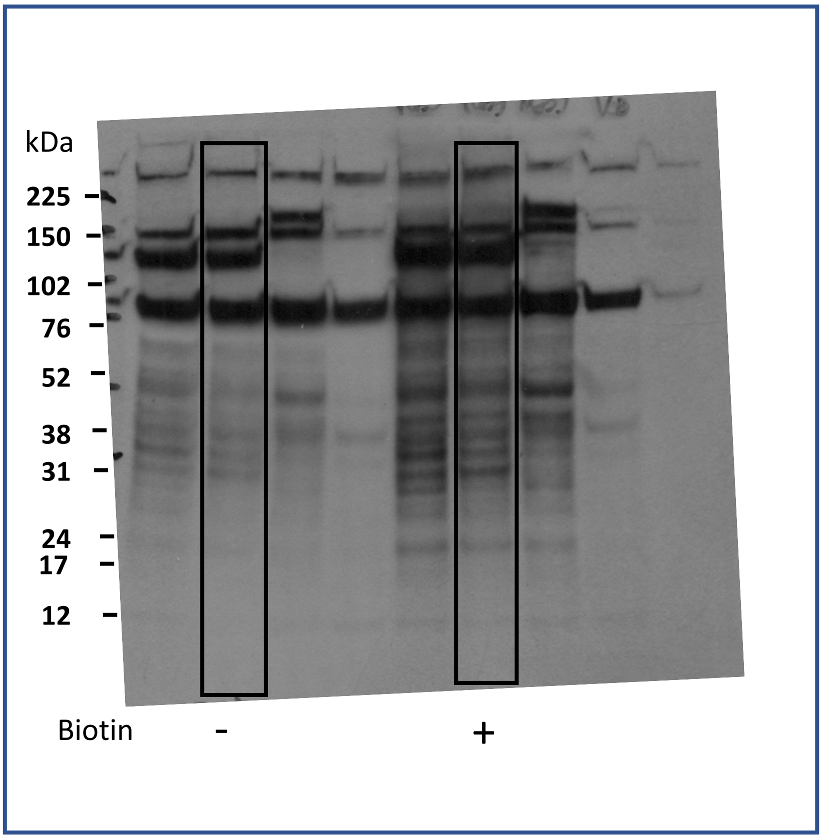

Figure 4A

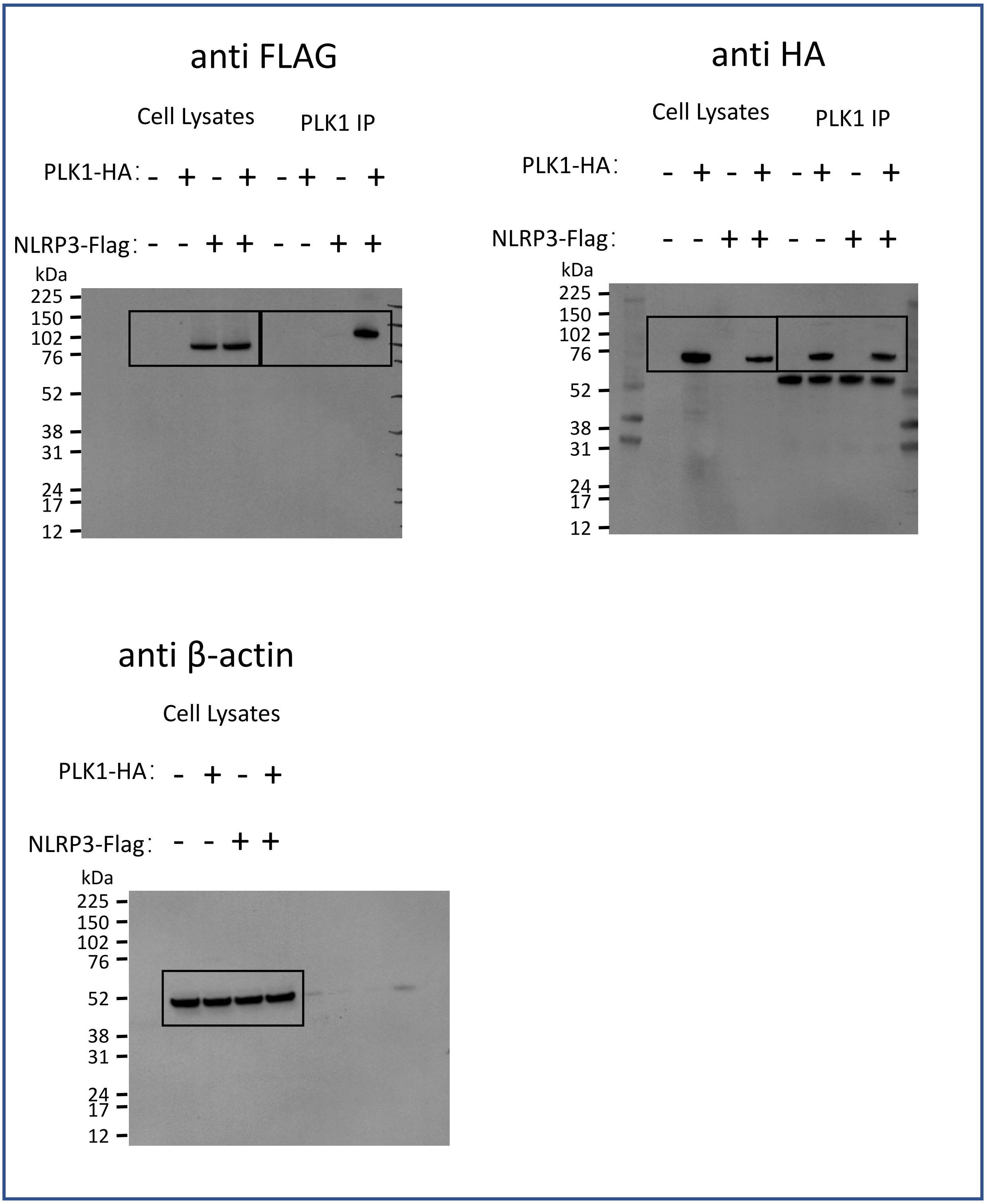
anti FLAG
Cell Lysates
PLK1 IP
PLK1-HA: - + - + - + - +
NLRP3-Flag: - - + + - - + +
kDa
225
150
102
76
52
38
31
24
17
12
anti HA
Cell Lysates
PLK1 IP
PLK1-HA: - + - + - + - +
NLRP3-Flag: - - + + - - + +
kDa
225
150
102
76
52
38
31
24
17
12
anti β-actin
Cell Lysates
PLK1-HA: - + - +
NLRP3-Flag: - - + +
kDa
225
150
102
76
52
38
31
24
17
12

Figure 4B

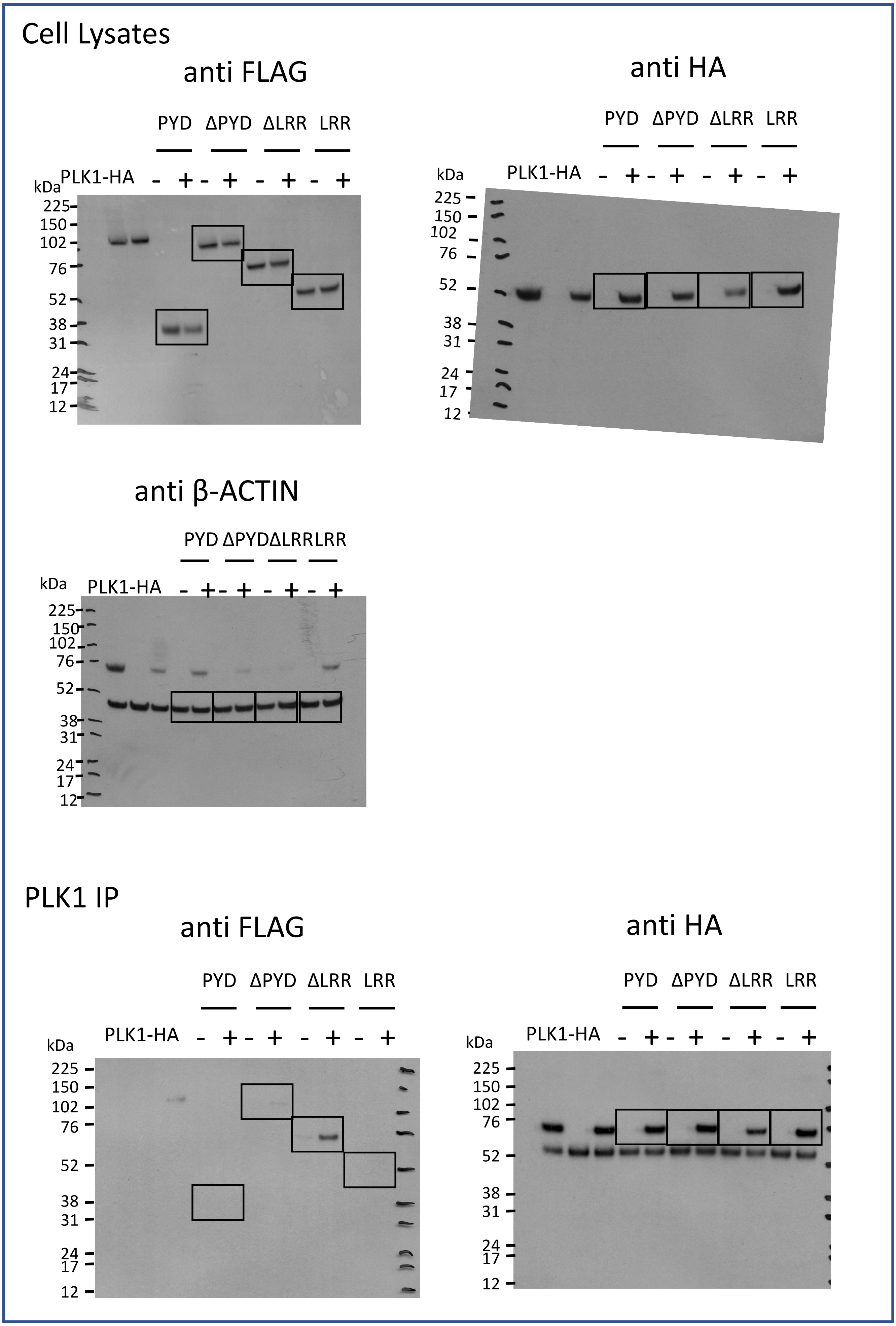

Cell Lysates
anti FLAG
PYD ΔPYD ΔLRR LRR
kDa PLK1-HA - + - + - + - +
225 150 102 76 52 38 31 24 17 12
anti HA
PYD ΔPYD ΔLRR LRR
kDa PLK1-HA - + - + - + - +
225 150 102 76 52 38 31 24 17 12
anti β-ACTIN
PYD ΔPYD ΔLRR LRR
kDa PLK1-HA - + - + - + - +
225 150 102 76 52 38 31 24 17 12
PLK1 IP
anti FLAG
PYD ΔPYD ΔLRR LRR
kDa PLK1-HA - + - + - + - +
225 150 102 76 52 38 31 24 17 12
anti HA
PYD ΔPYD ΔLRR LRR
kDa PLK1-HA - + - + - + - +
225 150 102 76 52 38 31 24 17 12

Figure 4C

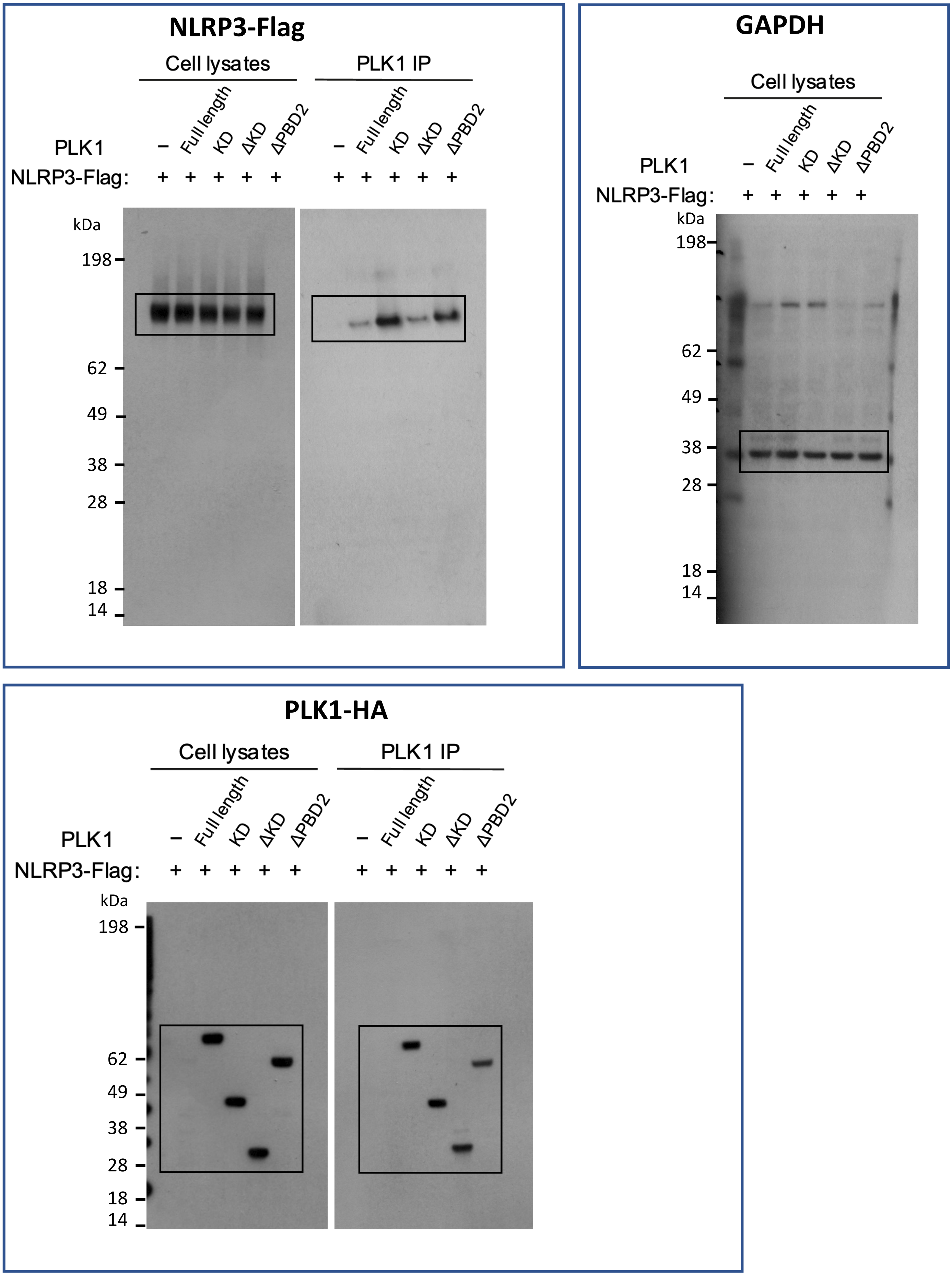
NLRP3-Flag
Cell lysates
PLK1 IP
PLK1
– Full length KD ΔKD ΔPBD2
– Full length KD ΔKD ΔPBD2
NLRP3-Flag: + + + + + + + + + +
kDa
198
62
49
38
28
18
14
GAPDH
Cell lysates
PLK1
– Full length KD ΔKD ΔPBD2
NLRP3-Flag: + + + + +
kDa
198
62
49
38
28
18
14
PLK1-HA
Cell lysates
PLK1 IP
PLK1
– Full length KD ΔKD ΔPBD2
– Full length KD ΔKD ΔPBD2
NLRP3-Flag: + + + + + + + + + +
kDa
198
62
49
38
28
18
14

Figure 5F

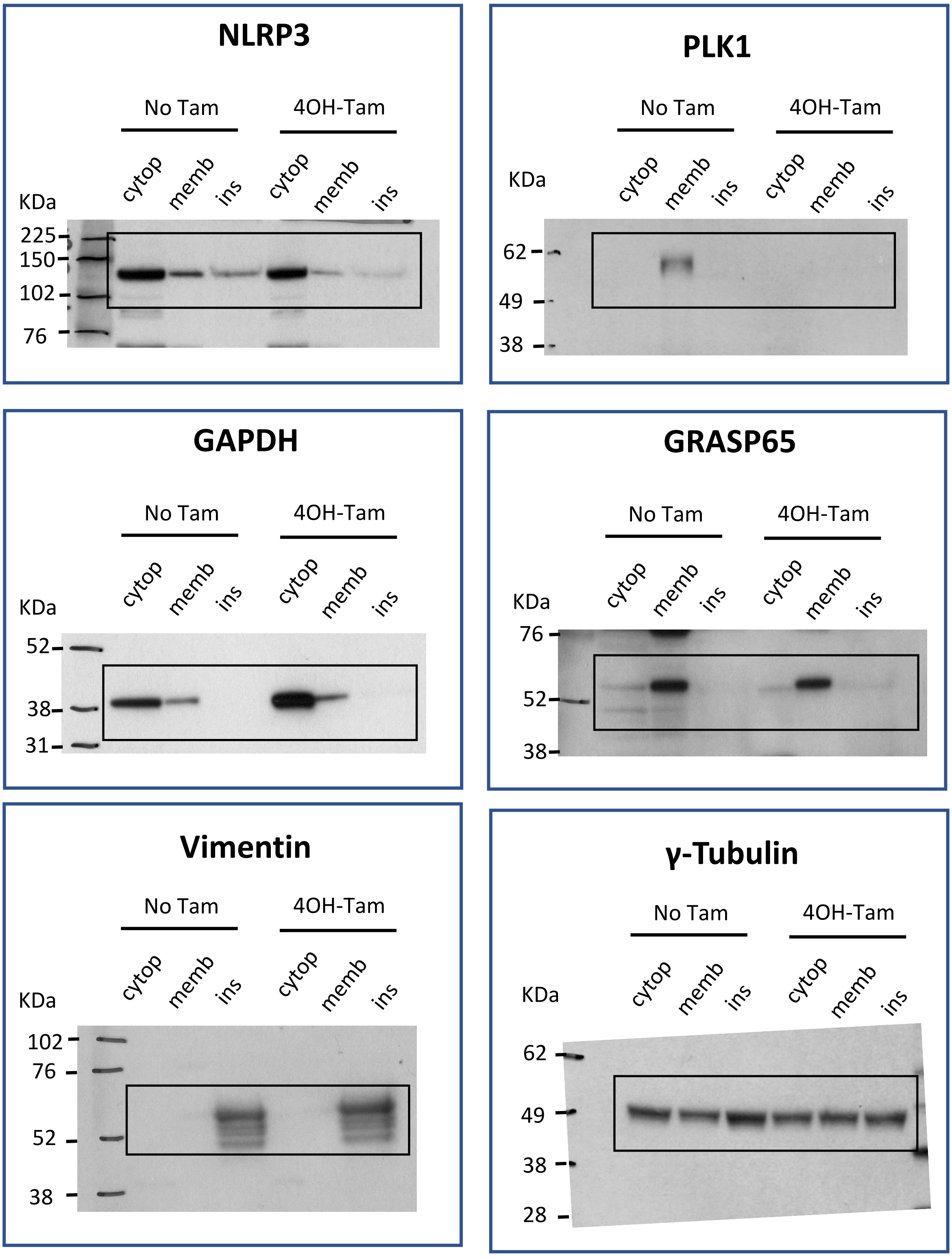
NLRP3
No Tam
4OH-Tam
cytop
memb
ins
cytop
memb
ins
KDa
225
150
102
76
PLK1
No Tam
4OH-Tam
cytop
memb
ins
cytop
memb
ins
KDa
62
49
38
GAPDH
No Tam
4OH-Tam
cytop
memb
ins
cytop
memb
ins
KDa
52
38
31
GRASP65
No Tam
4OH-Tam
cytop
memb
ins
cytop
memb
ins
KDa
76
52
38
Vimentin
No Tam
4OH-Tam
cytop
memb
ins
cytop
memb
ins
KDa
102
76
52
38
γ-Tubulin
No Tam
4OH-Tam
cytop
memb
ins
cytop
memb
ins
KDa
62
49
38
28

Figure S3E

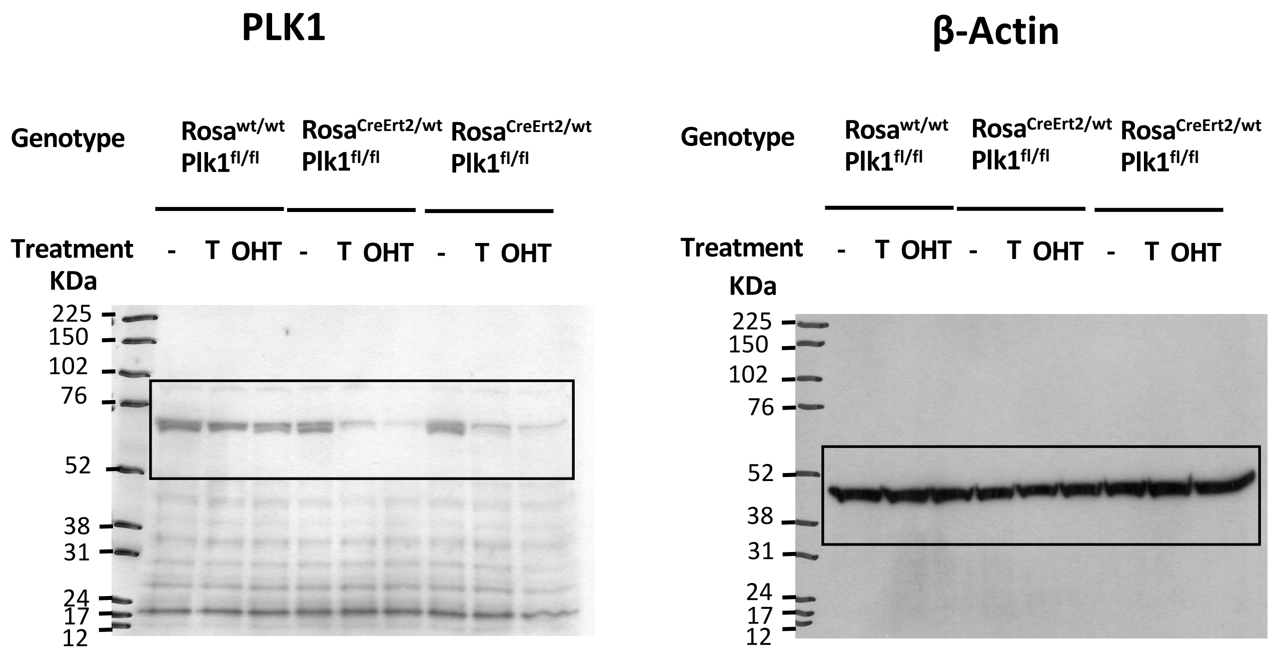

Figure S3K

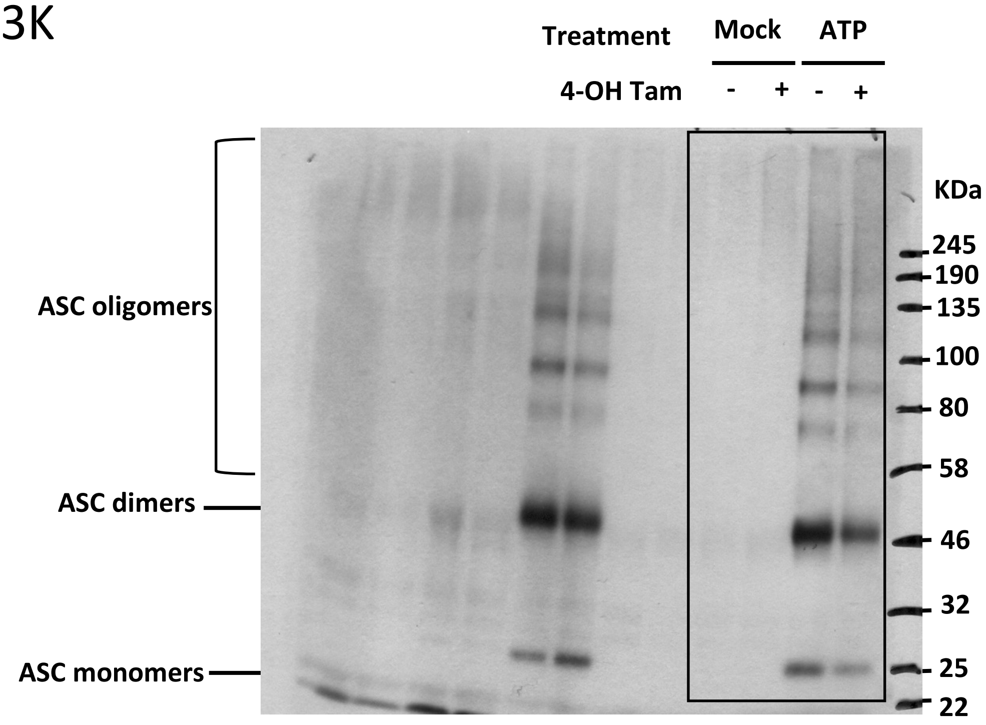

Supplement: Supplemental data [file jci-133-162129-s188.pdf]
